# Chronic bee paralysis virus exploits host antimicrobial peptides and alters gut microbiota composition to facilitate viral infection

**DOI:** 10.1093/ismejo/wrae051

**Published:** 2024-03-22

**Authors:** Yanchun Deng, Sa Yang, Li Zhang, Chenxiao Chen, Xuefen Cheng, Chunsheng Hou

**Affiliations:** Institute of Bast Fiber Crops, Chinese Academy of Agricultural Sciences, Changsha 410205, China; Institute of Apicultural Research, Chinese Academy of Agricultural Sciences, Beijing 100193, China; Graduate School of Chinese Academy of Agricultural Sciences, Beijing 100081, China; Institute of Apicultural Research, Chinese Academy of Agricultural Sciences, Beijing 100193, China; Institute of Bast Fiber Crops, Chinese Academy of Agricultural Sciences, Changsha 410205, China; Institute of Bast Fiber Crops, Chinese Academy of Agricultural Sciences, Changsha 410205, China; Institute of Bast Fiber Crops, Chinese Academy of Agricultural Sciences, Changsha 410205, China

**Keywords:** CBPV, antimicrobial peptide, gut microbiota, bloated abdomen, honey bees

## Abstract

The significance of gut microbiota in regulating animal immune response to viral infection is increasingly recognized. However, how chronic bee paralysis virus (CBPV) exploits host immune to disturb microbiota for its proliferation remains elusive. Through histopathological examination, we discovered that the hindgut harbored the highest level of CBPV, and displayed visible signs of damages. The metagenomic analysis showed that a notable reduction in the levels of *Snodgrassella alvi* and *Lactobacillus apis*, and a significant increase in the abundance of the opportunistic pathogens such as *Enterobacter hormaechei* and *Enterobacter cloacae* following CBPV infection. Subsequent co-inoculation experiments showed that these opportunistic pathogens facilitated the CBPV proliferation, leading to accelerated mortality in bees and exacerbation of bloated abdomen symptoms after CBPV infection. The expression level of antimicrobial peptide (AMP) was found to be significantly up-regulated by over 1000 times in response to CBPV infection, as demonstrated by subsequent transcriptome and quantitative real-time PCR investigations. In particular, through correlation analysis and a bacteriostatic test revealed that the AMPs did not exhibit any inhibitory effect against the two opportunistic pathogens. However, they did demonstrate inhibitory activity against *S. alvi* and *L. apis*. Our findings provide different evidence that the virus infection may stimulate and utilize the host’s AMPs to eradicate probiotic species and facilitate the proliferation of opportunistic bacteria. This process weakens the intestinal barrier and ultimately resulting in the typical bloated abdomen.

## Introduction

The honey bee, being a social insect, serves as an appropriate model organism for studying the gut microbiota in research [1]. Similar to other invertebrates, the gut microbiota of honey bees consists of host-specialized bacterial species, which are acquired through social interactions and environmental sources [1]. With a level of experimental control unavailable in human studies, honey bees offer an opportunity to understand the association between the microbiome and disease in a broader context [2]. This is facilitated by the relatively uncomplicated gut microbiota of bees, which is primarily composed of eight core bacterial species, constituting 95%–99% of the gut bacteria, in contrast to the more complex microbiota found in humans and other mammals. The most prevalent found bacterial strains in the gut of honey bees are typically *Lactobacillus* Firm5, *Lactobacillus* Firm4 (*Bombilactobacillus*), *Gilliamella apicola*, and *Snodgrassella alvi* [3–5]. In addition, honey bees are crucial pollinators of crops, especially the managed western honey bees (*Apis mellifera*) provide mobile pollination services to complement wild pollinators and account for 30%–50% of this ecosystem service [6]. Although *A. mellifera* is an important honey bee species in the global beekeeping industry [7], honey bee small RNA viruses have been considered as one of the main causes of honey bee colony decline [8–10]. Of these, chronic bee paralysis virus (CBPV) is one of the most prevalent viruses worldwide, presents in more than 75% of dead adult bees from *A. mellifera* apiaries in China [11]. However, limited information regarding the pathogenic mechanism of CBPV in honey bees has been reported thus far.

CBPV, as an unclassified segment RNA virus, consists mainly of two segments, RNA 1 and RNA 2. Its genome shares the similarities with the *Nodaviridae* and *Tombusviridae* families in a non-enveloped anisometric capsid [12]. CBPV can infect individuals at all developmental stages of bee and can be transmitted horizontally in a hive through contact between healthy and diseased bees [13]. CBPV spread via a variety of pathways within the hive, including fecal, oral, mechanical, and even vertical transmission through the queen [14, 15]. There has recently been an exponential rise in the incidence and viral loads of CBPV in Asia, Europe, and North America due to parasite coinfection or agrochemical exposure [14, 15]. Some studies have confirmed CBPV can result in adult bees displaying two typical symptoms: one is called as “hairless black syndrome,” in which infected individuals become nearly hairless and dark [14, 15]. The other characteristic symptom is dysentery that cannot be excreted, known as bloated abdomen [16]. Although several studies have recently focused on pathogenic mechanism of CBPV, including the assembly of the infection clone and impacts on host syrup consumption [14–17], there is little data on how CBPV infection evades host defenses and induces the typical symptom of bloated abdomen, as well as the tissue tropism. Is the gut microbiota responsible for this bloated abdomen? It appears that it must get past the innate immune system’s initial line of defense before causing typical symptoms.

For insects, innate immunity is primarily activated by the development of melanization and the generation of antimicrobial peptides (AMPs) [18]. AMPs have been shown to be one of the effectors in eliminating pathogenic bacteria and maintaining gut microbiota balance [19, 20]. The honey bee Toll/Imd signaling pathway plays a key role in resistant viral infection such as deformed wing virus (DWV) and Israeli acute paralysis virus (IAPV) [21, 22]. Recently, it was found that CBPV not only significantly induced the expression of AMPs [23], but also triggered the serine proteases cascades [24]. Based on the recent studies on the variations in the spatial distribution of gut microbiota and AMPs, the up-regulated AMPs may decrease the abundance of symbiotic bacteria [25]. This finding raises the question of whether there is a connection between AMPs, CBPV infection and the composition of gut microbiota.

A growing body of research has demonstrated the significant role of the gut microbiota in modulating the host immune response to combat viral infection in vertebrate [26–30]. Similarly, the gut microbiota have been found to play an important role in the antiviral defense of honey bee [31]. Our previous results also showed that tetracycline treatment decreased bee survival following IAPV infection and increased the susceptible of *Apis cerana* to IAPV infection [32]. Furthermore, a recent study revealed that *S. alvi*, a core bacteria, could stimulate the expression of *apidaecin* and *hymenoptaecin*, thereby elevating bee survival upon challenge with DWV [33]. These findings emphasize the importance of the gut microbiota in protecting against viral infections. However, recent studies have also showed that certain gut bacteria can enhance the replication, transmission, and pathogenesis of various enteric viruses [34–37]. It has been reported that enteric viruses could bind to bacteria surface polysaccharides, thereby improving cell attachment and movement, potentially promoting virus transmission and infection [37]. Despite honey bees serving as an excellent model organism for studying gut microbiota [1, 38, 39], there is a paucity of research on how CBPV infection changes the composition of the gut microbiota in honey bees. It is noteworthy that there is limited understanding of how CBPV influences the gut microbiome to induce bloated abdomen in honey bees.

In the current study, tetracycline treatment slightly reduced CBPV proliferation and effectively relieved symptoms of bloated abdomen, whereas treatment with two opportunistic pathogens slightly increased the CBPV proliferation and markedly aggravated the symptoms of bloated abdomen. The expression levels of AMPs (*defensin1* and *hymenoptaecin*) were significantly negatively correlated with the abundance of core probiotic genus *Snodgrassella* and *Lactobacillus*, but extremely positively with the abundance of opportunistic pathogens of the genus *Enterobacter* spp. The bacteriostatic test also showed that the two AMPs exhibited slight and obvious inhibitory effect against *S. alvi* and *Lactobacillus apis*, respectively, but not against the two opportunistic pathogens *Enterobacter hormaechei* and *Enterobacter cloacae*. These findings not only demonstrated a different mechanism of gut microbiota suppression by CBPV infection, which induces the production of AMPs that target the core probiotic species to facilitate viral infection, but also indicated that maintaining gut microbiota homeostasis is a potential antiviral drug candidate.

## Materials and methods

### Samples

Three honey bee (*A. mellifera*) colonies were collected from Guangdong Institute of Applied Biological Resource, Guangzhou, China. These honey bees were seemingly identified as healthy, and were free from bacterial diseases such as American foulbrood and European foulbrood, and common honey bee viruses following the method of previous study with the special primers (Table S1) [11, 40]. They were also free from *Varroa* mites and fungal diseases (*Nosema*, Chalkbrood and Stonebrood) under a microscope. About 30 newly emerged healthy honey bees were transferred into the standard wooden cage (8 cm × 6 cm × 12 cm) as one repeat (three repeats for each group), and all the cages were kept in an artificial climate incubator (MGC-800HP, shanghai, China) with 2 ml 50% sucrose solution provided daily as previously described [11].

### Infection on honey bees with CBPV

**Figure 1:**
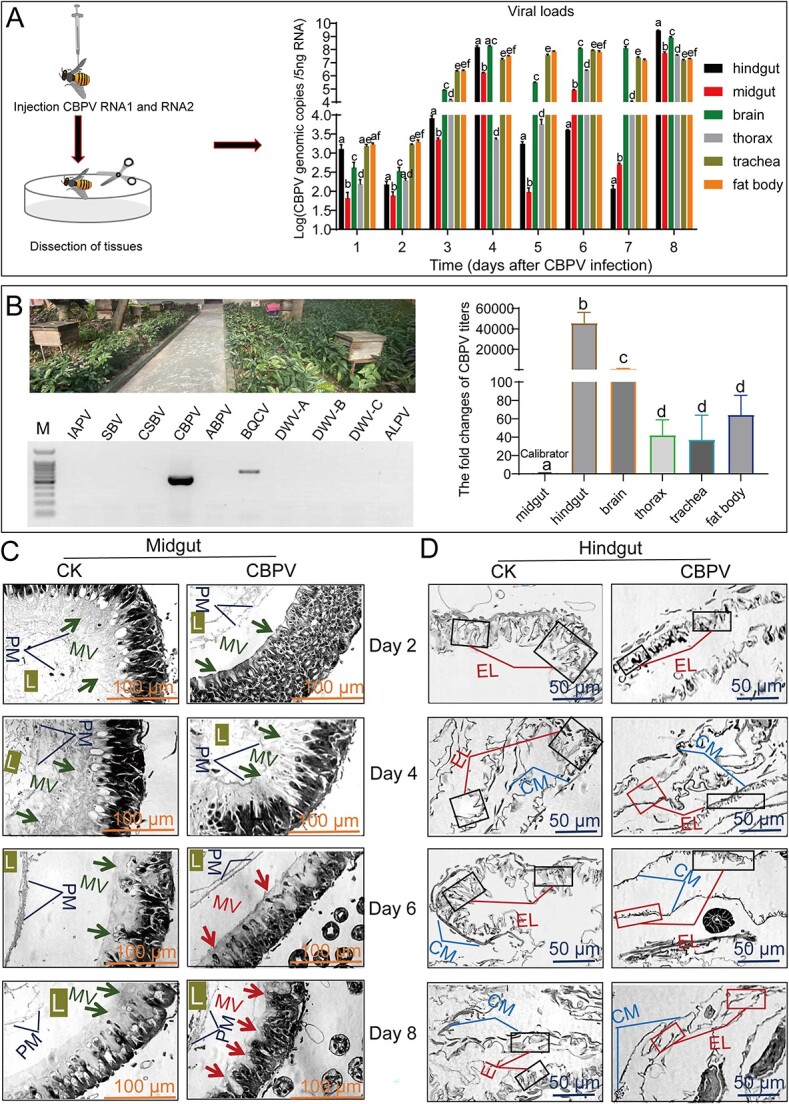
Viral titers in different tissues of honey bees injected with the infectious clone of CBPV at various time points and the damage caused by CBPV infection in different tissues of *A. mellifera*. (A) Quantification of CBPV titers in six tissues: trachea, fat body, hindgut, thorax, brain, and midgut of honey bees at Days 1, 2, 3, 4, 5, 6, 7, and 8. (B) Quantification of CBPV titers on trachea, fat body, hindgut, thorax, brain, and midgut of bees from naturally infected predominantly with CBPV in field. (C) The histology of midgut of honey bee after CBPV infection at Days 2, 4, 6, and 8. (D) The histology of hindgut of honey bee after CBPV infection at Days 2, 4, 6, and 8. L, lumen; PM, peritrophic membranes; MV, microvilli; CM, circular muscle.

Due to the lack of CBPV cell culture systems in vitro, the construction of infectious clones has become a feasible way to study the pathogenesis of honeybee viruses, as our previous study led to the construction of one CBPV infectious clone [16]. Approximately 30 newly emerged honey bees were transferred into the standard wooden cage (8 cm × 6 cm × 12 cm), constituting one trial (with three trials conducted for each group). The cages were then kept in an artificial climate incubator (MGC-800HP, Shanghai, China), with a daily provision of 2 ml 50% sucrose solution. These honey bees were subsequently administrated 2 μL of purified synthetic CBPV RNA1 and RNA2 (approximately 1 × 10^12^ genome copies) via a Hamilton syringe (Hamilton, Switzerland) into the thorax (702) (Fig. 1A). The control group received injection of PBS. The entire process of injecting CBPV RNA or PBS to all honey bees was completed in 2 hours. Following the injection, these groups were transferred to an incubator set at 30°C/60% RH, and dead honey bees were observed and recorded daily. To minimize the effect of bee sampling on mortality results during the experiment, we set up additional five parallel experiments, with one bee sampled from each of the five groups at a time for RNA extraction, tissue dissection, and DNA extraction, respectively. In addition, 30 honey bee workers naturally infected with CBPV were obtained from the Institute of Zoology, Guangdong Academy of Sciences (Guangzhou, China), and subsequently underwent tissues dissection for further experiments.

### Tissue dissection, histopathological examination, DNA extraction, and metagenome sequencing

To investigate CBPV proliferation in different tissues of honey bees, five honey bees were collected from each of group maintained in an incubator from day one to eight postinfection with CBPV. Tissues (trachea, fat body, hindgut, thorax, brain, and midgut) dissection was performed following the method of our previous study [32]. After that, histopathological analysis of gut of honey bees was performed as described previously [41].

Genomic DNA of gut microbial was obtained from five honey bees that were either injected with PBS or infected with CBPV. The DNA extraction process was carried out on frozen gut samples using a total DNA extraction kit supplied by Hangzhou Foreal Nanotechnology (Hangzhou, China) according to the manufacturers instructions. Subsequently, the microbial genomic DNA extracted was subjected to sequencing using a HiSeq System (Illumina) at Novogene in Beijing, China.

### RNA extraction, quantitative real-time PCR analysis, and RNA-seq

To assess the proliferation level of CBPV in various tissues, the total RNA from brain, muscle, midgut, fatty body, trachea, and hindgut sample of bees infected with CBPV from Days 1 to 8 was extracted using RNAprep pure Micro kit (TIANGEN, Beijing, China) in accordance with the manufacturer’s instructions. In addition, the total RNA from whole body sample of five honey bees injected with PBS or CBPV RNA was extracted using the TRIzol Kit (Ambion, Life Technologies, USA) following the manufacturers guidelines on Days 1, 3, 5, and 7. Subsequently, the replication level of CBPV was quantified through quantitative real-time PCR with the specific primers (Table S2), and the standard curves for absolute quantification of CBPV genes and immune genes was presented in Table S3. To enhance our understanding of the immune response following infection, we subsequently examined the genes that were expressed differentially after CBPV infection at various time points (0, 24, 48, 72, and 96 hours) through transcriptome analysis and qPCR. The extracted RNA of each sample of PBS and CBPV-injected adult honey bees was used for library construction and RNA-seq at Novogene (Beijing, China). The interaction networks of corresponding proteins of the target genes were predicted using STRING (https://cn.string-db.org/) and weighted gene co-expression network analysis (WGCNA) [42]. qPCR analysis and RNA sequencing was described in detail in support information.

### Tetracycline or the opportunistic pathogens treatment in CBPV-infected honey bees

Newly emerged honey bees were randomly assigned to receive tetracycline at a concentration of 500 μg/ml to eliminate the majority of gut bacterial populations [2]. A detailed description of the protocol can be found in the supplementary information.

For the opportunistic pathogens treatment, gut bacteria strains from honey bee were initially isolated using Luria-Bertani (LB) or de Man Rogosa Sharpe (MRS) broth, respectively (Fig. S10), and then identified through PCR analysis using the 16S rRNA genes. Subsequently, the newly emerged honey bees were randomly assigned to consume a mixture of *E. cloacae*, *E. hormaechei*, and 50% sugar solution, with the sterilized 50% sugar solution supplied as a control. The mortality was monitored daily, and viral proliferation was tested using qPCR.

### Expression and purification of AMPs proteins, and analysis of the antibacterial activity of AMPs

To obtain soluble Defensin1 and Hymenoptaecin proteins in vitro, plasmids of pET28a-Defensin1 and pET28a-Hymenoptaecin were constructed according to the manufacturer’s manuals (Table S1). The inhibitory activity of AMPs induced by CBPV infection against core probiotic species and opportunistic pathogens was assessed using the Oxford cup method and growth curves test.

### Statistical analysis

The average survival rate (the Log-rank (Mantel-Cox) test) and correlation analysis (two-tailed) among the different treatment groups were statistically tested using GraphPad Prism 8 (GraphPad Software Inc., San Diego, CA, USA). The mean values of standard deviation (SD) were calculated using Microsoft Excel. Spearman correlation analysis was examined in GraphPad Prism 8. The differences of gene expression levels were assessed using Tukey’s multiple comparison in GraphPad Prism 8. Volcano Plot and the column graph were finished by GraphPad Prism 8. The number of bacteria was determined using absolute quantification and relative quantification to determine the core species (core species/the total bacterial population).

## Results

### Hindgut harbors the highest CBPV abundance and results in bloated abdomen

As previously described [15, 16], honey bees infected with the CBPV infectious clone displayed apparent paralysis and bloated abdomen. The mortality of CBPV-infected honey bees gradually increased from Days 1 to 7 compared to the control groups (39%, *P* < 0.01) (Fig. S1A). However, the mortality of bees after CBPV infection reached 9% at Day 5. Likewise, we observed a gradual rise in CBPV titers from Days 1 (10^3^) to 4 (10^7^ genome copies), but dropped to 10^6^ genome copies at Day 5 (Fig. S1B).

To explore the reason behind the bloated abdomen caused by CBPV, we initially studied the growth of CBPV in various tissues, particularly the gut. Our results showed that CBPV titers steadily increased from Days 1 to 4 in the major tissues, but then sharply dropped by over 10^5^ times in the midgut and hindgut at Day 5 compared to Day 4 (Fig. 1A). In fact, the viral loads of CBPV significantly varied across different tissues, and were in the following order from the highest to lowest: brain (1.82 × 10^8^) > hindgut (1.55 × 10^8^) > fat body (3.28 × 10^7^) > trachea (1.79 × 10^7^) > midgut (1.73 × 10^6^) > thorax (2.29 × 10^3^) at Day 4 after CBPV infection (Fig. 1A). As the infection went on, the hindgut harbored the highest titers (2.81 × 10^9^) at Day 8, followed by the brain (8.16 × 10^8^), midgut, thorax, fat body, and trachea (Fig. 1A). Similar results were obtained in bees with CBPV infection under natural conditions in the field: hindgut > brain > fat body > thorax > trachea > midgut (Fig. 1B). These results indicated that no matter which way infection, CBPV was mainly distributed in hindgut and brain.

To verify the sites of injury caused by CBPV, as previously described about the symptoms of bloated abdomen [16], histopathological analysis of the honey bee midgut and hindgut was performed using a Leica DFC280 light microscope at Days 2, 4, 6, and 8. CBPV infection caused variable degrees of damage in these two tissues at Days 4, 6, and 8 (Fig. 1C and D). Damaged microvilli were seen in the midgut of CBPV-infected honey bees from Days 6 to 8 (Fig. 1C). Moreover, CBPV obviously caused cell degradation in the hindgut from Day 4, with more cells being shed as the viral infection progressing (Fig. 1D), and viral load apparently altered (Fig. 1A). These findings suggested that the hindgut harbored the highest level of CBPV and the main site of the typical symptom of CBPV-induced bloated abdomen was the gut.

### CBPV infection significantly alters gut microbiota composition

**Figure 2:**
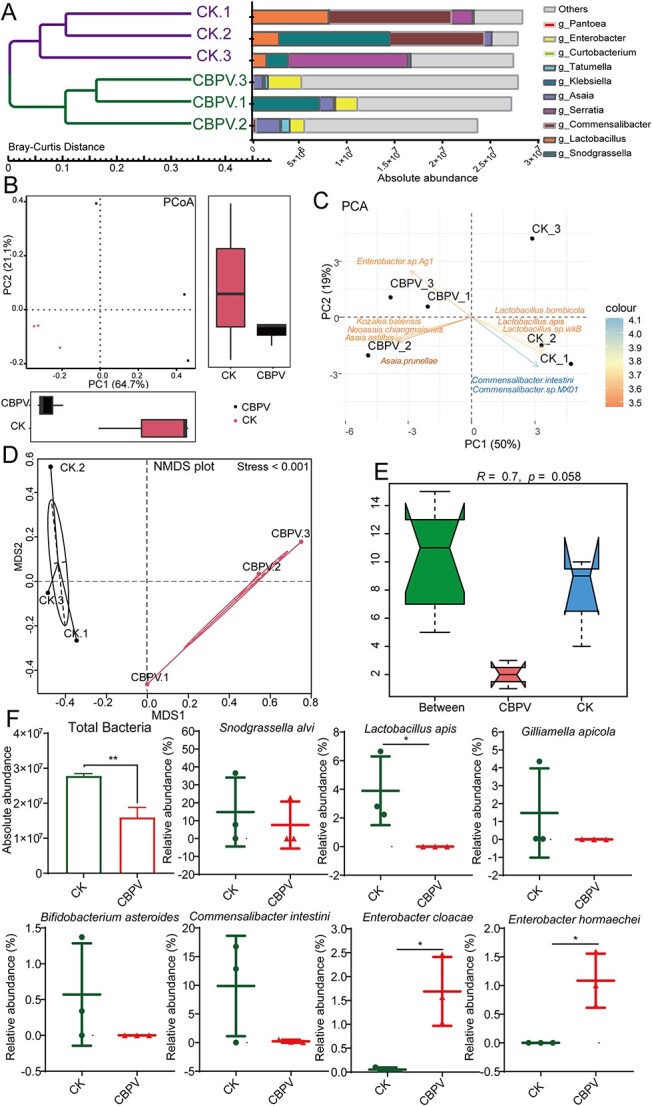
Metagenome sequencing analysis showed that viral infection affects the gut microbiota composition in *A. mellifera* at Day 5. (A) The UPGMA analysis showed the absolute abundance of gut bacteria species in healthy and CBPV-infected honey bees at the genus level. (B) PCoA analysis exhibited a variation in microbial diversity between healthy and CBPV-infected honey bees. (C) PCA analysis exhibited the difference in microbial diversity between healthy and CBPV-infected honey bees. (D) NMDS analysis exhibited the difference in microbial diversity between healthy and CBPV-infected honey bees. (E) ANOSIM analysis exhibited variation in microbial diversity between healthy and CBPV-infected honey bees. (F) The column graph depicted the absolute abundance of gut bacteria species, and the dot plot displayed the relative abundances of gut microbiota core species in healthy and CBPV-infected honey bees. Each group has three replicates. Significant variations in each gene from the different groups are indicated by asterisks. ^*^P < 0.05.

The relative abundances of gut bacteria species in healthy and CBPV-infected honey bees were modified at the phylum, class, order, and family levels (Fig. S2), indicating that viral infection altered the bacteria communities in *A. mellifera*. Especially, CBPV infection significantly increased the relative abundance of sphingomonadaceae, exceeding 100 times in comparison to the healthy group (*P* < 0.01) (Fig. S2). Venn diagrams illustrating the presence of shared and unique taxa at the family and genus level (with relative abundance > 0.01%) in both the control and CBPV-infected groups suggested that the genus *Bifidobacterium* spp. was exclusively present in healthy honey bees, whereas the genus *Staphylococcus* spp. was unique in CBPV-infected honey bees (Fig. S3). The examination of alpha diversity, including the ace, chao, and Simpson index, with the exception of the Shannon index, revealed that the microbial diversity in the CBPV group was comparatively reduced in comparison to the healthy group (Fig. S4). UPGMA analysis using Bray-Curtis distance and Principal Coordinates Analysis (PCoA) analysis both showed that CBPV infection resulted in considerable variation in the absolute abundance of gut bacteria at the genus level (Fig. 2A and B). PCA analysis suggested that healthy honey bees had higher levels of *L. apis*, whereas CBPV-infected honey bees had higher level of *Enterobacter* (Fig. 2C). NMDS analysis (stress < 0.001) and ANOSIM analysis (*R* = 0.70, *P* < 0.06) further demonstrated that CBPV infection induced the substantial variations in microbial diversity (Fig. 2D and E). Likewise, CBPV infection induced a decrease in the size of the gut bacteria (*P* < 0.01) (Fig. 2F), showing a significant decrease in the absolute abundance (the total bacterial 16S rRNA gene copies) of gut bacteria from 2.8 × 10^7^ to 1.5 × 10^7^ following CBPV infection. The use of multinomial regression allows for the direct estimation of relative differentials [43], particularly in the total gut bacteria, where the core species composition at the relative abundance level exhibited more pronounced changes after CBPV infection (Fig. 2F). Specifically, the relative abundances of the common core species *S. alvi*, *L. apis*, *G. apicola*, *Bifidobacterium asteroides*, *Lactobacillus melliventris*, and symbiotic bacteria *Commensalibacter intestini* decreased to 7.6%, 0.0001%, 0.006%, 0.00002%, 0.005%, and 0.11% in the CBPV-treated group, compared to the control group with relative abundances of 14.8%, 3.9%, 1.5%, 0.57%, 0.88%, and 9.7%, respectively (Fig. 2F, Table S4). Moreover, CBPV-infected group were further distinct from the healthy group for having greater numbers of opportunistic pathogens *E. cloacae* (2.51%) and *E. hormaechei* (1.1%), compared to the control group (both <0.06%) (*P* < 0.05) (Fig. 2F, Table S4).

### The increased opportunistic pathogens bacteria promote CBPV infection

The obvious change in the gut microbiota motivated us to further examine whether and how the gut microbiota are closely related to viral infection. We used KEGG pathway analysis and carbohydrate-active enzymes (CAZymes) function annotation to better understand the link between the bacteria community and viral infection (Fig. S5). The absolute abundance of gut microbiota associated with metabolism of amino acid was significantly up-regulated (*P <* 0.01) in CBPV-infected *A. mellifera*, whereas the absolute abundance of gut microbiota associated with lipid metabolism was significantly decreased (*P* = 0.013) (Fig. S5A, Table S5). In addition, the relative abundance of gut microbiota related to cell motility (*P* < 0.07) and membrane transport (*P* < 0.5) was up-regulated after CBPV infection (1.7%, 4.66%) compared to the control (0.33%, 4.2%) (Fig. S5B, Table S5)*.* These results suggested that CBPV infection accelerated cell motility and membrane metabolism of gut microbiota to facilitate its survival. Furthermore, the relative abundances of gut microbiota related to CAZymes, such as carbohydrate esterases, were also found to be increased following CBPV infection (*P* < 0.02) (Fig. S5C, Table S6), suggesting that altered gut microbiota may contribute to provide additional energy for viral infection.

**Figure 3:**
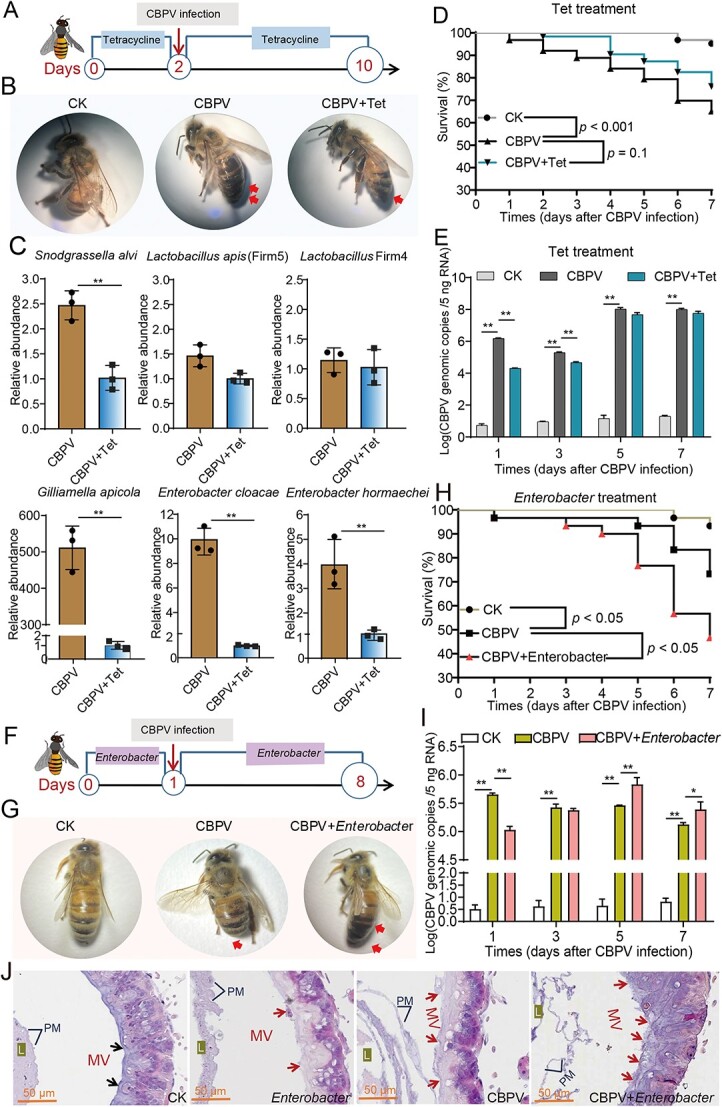
The effect of tetracycline or opportunistic pathogens treatment on gut microbiota, CBPV loads and physiology of *A. mellifera*. (A) Schematic illustration of the tetracycline experimental design. (B) The photo of the newly emerging bees after CBPV infection and tetracycline treatment. (C) The relative abundances of gut microbiota were quantified by qPCR with specific bacterial species, and normalized by 16S rRNA gene. (D) Survival rates of the CBPV-infected newly emerging bees with and without tetracycline treatment. (E) The effect of tetracycline on CBPV genomic copies in the newly emerging bees after viral infection. (F) Schematic illustration of the opportunistic pathogens experimental design. (G) The photo of the newly emerging bees after CBPV infection and opportunistic pathogens treatment. (H) Survival rates of the CBPV-infected newly emerging bees after opportunistic pathogens treatment. (I) The effect of opportunistic pathogens on CBPV genomic copies in the newly emerging bees after viral infection. (J) The histology of gut of the newly emerging bees after CBPV infection and opportunistic pathogens treatment. Asterisks indicate significant differences between groups. ^*^P < 0.05, ^**^P < 0.01. L, lumen; PM, peritrophic membranes; MV, microvilli.

It has been reported that the majority of enterobacteria showed the strongest antibiotic resistance to ampicillin and cephalothin but was sensitive to tetracycline and kanamycin [44]. To further investigate the possible aid of this specific gut bacteria for CBPV proliferation, tetracycline was fed to CBPV-infected honey bees to decimate microbes including pathogenic *E. hormaechei* and *E. cloacae* (Fig. 3A). The representative images showed that CBPV infection caused obvious bloated abdomens at Day 10, whereas tetracycline treatment alleviated these typical symptoms caused by CBPV infection (Fig. 3B). As expected, treatment with tetracycline not only significantly reduced the abundance of the core specie *S. alvi* (by 1.4-fold, *P* < 0.01), *Lactobacillus* Firm4 (by <0.5-fold) and *G. apicola* (by 500-fold, *P* < 0.01), but also markedly decreased the abundances of opportunistic pathogens *E. cloacae* (by 3-fold, *P* < 0.01) and *E. hormaechei* (by 8-fold, *P* < 0.01) (Fig. 3C). Furthermore, compared to the control group (95%), the survival rate in the CBPV infection group was 56%, and the CBPV and tetracycline treatment group had a survival rate of 76% at Day 7, indicating that tetracycline slightly reduced the mortality of honey bees after CBPV infection (Fig. 3D). The CBPV loads in the tetracycline-CBPV treatment group was significantly lower than that in the CBPV treatment alone (*P* < 0.01), but significantly decreased by about 4-fold only at Days 1 and 3 (Fig. 3E).

The opportunistic pathogens treatment (the mixture of *E. cloacae* and *E. hormaechei*) was performed to further assess the impact of gut microbiota on CBPV proliferation (Fig. 3F). Our result showed that the opportunistic pathogens treatment aggravated the typical symptoms of bloated abdomens at Day 7 after CBPV infection (Fig. 3G). Compared to the control group (93%), the survival rate in CBPV infection group was 73%, and the CBPV and opportunistic pathogens treatment group had a survival rate of 47% at Day 7, indicating that opportunistic pathogens could significantly reduce mortality rate of honey bees after CBPV infection (*P* < 0.05) (Fig. 3H). In addition, the quantity of CBPV in the opportunistic pathogens group was significantly higher compared to the group receiving CBPV treatment alone (*P* < 0.01), showing a significantly increase of approximately 2-fold only on Days 5 and 7 (Fig. 3I). Additional histopathological examination revealed that the treatment of opportunistic pathogens caused minor damage to the gut microvilli in bees without the virus. This damage then escalated, resulting in thinner and disrupted gut microvilli in bees infected with CBPV at Day 7, compared to bees infected with CBPV alone (Fig. 3J). Taken together, these results further indicated that the opportunistic pathogens can provide more convenience to help CBPV infection in honey bees, and *E. cloacae* and *E. hormaechei* were the one of the main contributors to result in the bloated abdomen in honey bees after CBPV infection.

### AMPs induced by CBPV infection inhibited the growth of the core probiotics

**Figure 4:**
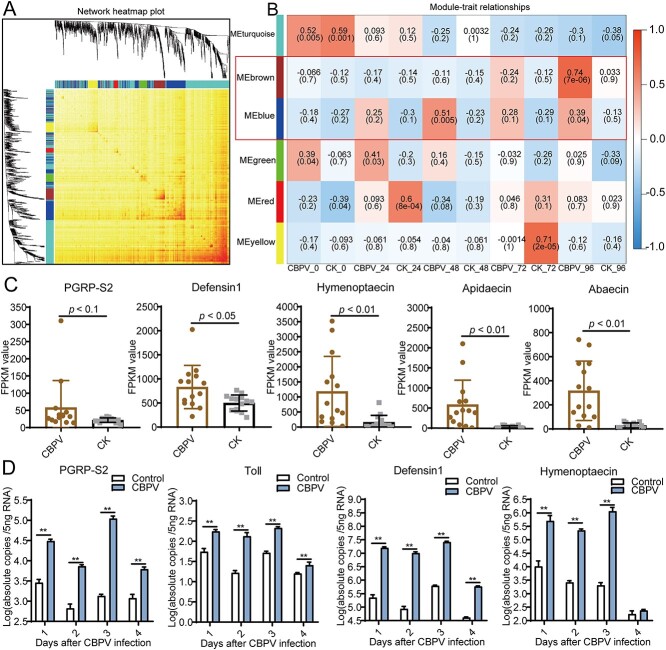
The AMPs were significantly induced after CBPV infection. (A) The co-expression modules of the expressed genes in healthy and CBPV-infected honey bees, and the clustering dendrogram of 30 samples. (B) The relationship of 10 traits and 6 modules. (C) The FPKM values of five immune genes, *peptidoglycan recognition protein S2* (*PGRP-S2*), *hymenoptaecin*, *defensin1*, *apidaecin*, and *abaecin*. (D) The expression levels of the immune genes in Toll/Imd pathway in the newly emerged CBPV-infected *A. mellifera* at Days 1, 2, 3, and 4. ^*^P < 0.05, ^**^P < 0.01.

To further gain insight into the relationship between the gut microbiota composition and the CBPV infection, we then investigated the differentially expressed genes after CBPV infection at hours 0, 24, 48, 72, and 96 using transcriptome analysis and qPCR. The transcriptome sequencing was utilized to acquire the Fragments Per Kilobase of transcript per Million mapped reads (FPKM) values of all expressed genes in bees following infection with CBPV at five different time points (Table S7). A co-expression network was constructed based on the gene expression matrix with WGCNA (Fig. 4A), and we obtained a total of 14 modules, the correlations between the featured genes of each module were presented in Figure 4B. The brown and blue modules were then found to have a high correlation with CBPV infection, and 388 genes were included in the two modules (Fig. 4B, Table S8). The function analysis for the 388 genes was further elaborated by STRING and the innate immune pathway was most significantly enriched (*P* < 0.0003), the immune genes including *peptidoglycan recognition protein S2* (*PGRP-S2*), *hymenoptaecin*, *defensin1*, *apidaecin*, and *abaecin* (Table S8). As we have seen, the expression levels of the genes *PGRP-S*2, *hymenoptaecin*, *defensin1*, *apidaecin*, and *abaecin* in the Toll/Imd signaling pathway were significantly up-regulated, especially the AMPs, the up-regulation fold was more than 100 times after CBPV infection (Fig. 4C) (*P* < 0.05). Simultaneously, the qPCR results also showed that the genes *PGRP-S2*, *hymenoptaecin*, and *defensin1* in the Toll/Imd pathway were all up-regulated more than three times after CBPV infection (Fig. 4D). Especially, the expression of *hymenoptaecin* and *defensin1* was up-regulated by at least 100 times (*P* < 0.01) (Fig. 4D). These results suggested that, AMPs, acting as specific effectors to regulate gut microbiota composition [25, 45], were significantly induced after CBPV infection in honey bees.

To confirm the link between AMPs and gut microbiota composition, we tested the expression levels of ten genes correlated with AMPs. Our results showed that tetracycline treatment not only led to a significant decrease in the abundance of gut microbiota bacteria (Fig. 3C), but also obviously reduced the expression levels of PGRP-S2, *Toll, relish,* and several AMPs such as *hymenoptaecin*, *apisimin*, *abaecin*, and *lysozyme* (Fig. S6). Subsequent analysis revealed a slight down-regulation in the expression level of *defensin1*, whereas the expression level of *hymenoptaecin* was significantly down-regulated by more than 2-fold (*P* < 0.05) following tetracycline treatment in the CBPV-infected honey bees (Fig. S7A and B). Conversely, treatment with opportunistic pathogens significantly promoted the expression level of *defensin1* and *hymenoptaecin* by more than 2-fold after CBPV infection on Days 3 and 5 (Fig. S7C and D).

**Figure 5:**
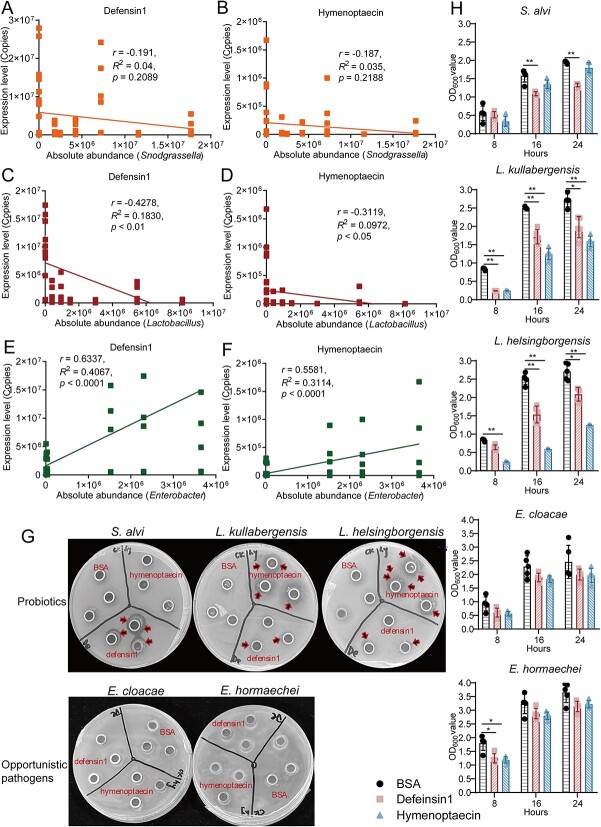
The inhibition activity of AMPs induced by CBPV infection against core probiotic species and opportunistic pathogens. (A) The correlation analysis between the expression level of *defensin1* and the absolute abundance of the genus *Snodgrassella*. (B) The correlation analysis between the expression level of *hymenoptaecin* and the absolute abundance of the genus *Snodgrassella*. (C) The correlation analysis between the expression level of *defensin1* and the absolute abundance of the genus *Lactobacillus*. (D) The correlation analysis between the expression level of *hymenoptaecin* and the absolute abundance of the genus *Lactobacillus*. (E) The correlation analysis between the expression level of *defensin1* and the absolute abundance of the genus Enterobacter. (F) The correlation analysis between the expression level of *hymenoptaecin* and the absolute abundance of the genus Enterobacter. (G) The representative LB/MRS plates showed the inhibition activity of Defensin1 or Hymenoptaecin against the gut microbiota. The core probiotic species (*S. alvi*, *L. kullabergensis*, *L. helsingborgensis*) and opportunistic pathogens (*E. cloacae*, *E. hormaechei*) were isolated from the gut of CBPV-infected *A. mellifera*, was spotted onto the LB/MRS plates and then incubated with BSA (0.5 mg/ml), Defensin1 (0.5 mg/ml), or Hymenoptaecin (0.5 mg/ml), culturing for 24 hours at 37°C. (H) The growth curves showed the inhibition activity of Defensin1 or Hymenoptaecin (0.02 mg/ml) against the gut microbiota.

We then combined our results in this study with the previous studies [5] and analyzed the correlation between the expression of AMPs and the abundance of gut microbes, including core probiotics (like *Snodgrassella* and *Lactobacillus*) and opportunistic pathogenic bacteria (such as *Enterobacter*). Spearman correlation analysis showed that the expression profiles of *defensin1* and *hymenoptaecin* were significantly negatively correlated with the absolute bacteria abundance after CBPV infection (*P* < 0.01) (Fig. S8). Especially, the comparative analysis showed that the expression levels of *defensin1* and *hymenoptaecin* had a negative correlation with the abundance of *Snodgrassella* (*r* = −0.19, *P* = 0.21; *r* = −0.19, *P* = 0.22), and were significantly negatively correlated with the abundance of *Lactobacillus* (*r* = −0.43, *P* < 0.01; *r* = −0.31, *P* < 0.05) (Fig. 5A–D). In contrast, the expression levels of *defensin1* and *hymenoptaecin* showed an extreme correlation with the abundance of opportunistic pathogenic bacteria *Enterobacter* (*r* = 0.63, *P* < 0.0001; *r* = 0.56, *P* < 0.0001) (Fig. 5E and F).

To further identify the inhibitory effect of AMPs on the core species, Defensin1 and Hymenoptaecinon protein were isolated using His Pur Ni-NTA Resin in vitro (Fig. S9). These isolated proteins were subsequently employed in an antibacterial activity assay conducted through the use of an Oxford cup. Our results showed that Defensin1 exhibited strongly antibacterial activity on core species Gram-negative *S. alvi* and Gram-positive *Lactobacillus kullabergensis* and *Lactobacillus helsingborgensis*, but not effective against Gram-negative opportunistic pathogenic bacteria *E. hormaechei* and *E. cloacae* (Fig. 5G). Likewise, Hymenoptaecin had significantly antibacterial activity on Gram-positive *L. kullabergensis* and *L. helsingborgensis*, but no antibacterial activity on *S. alvi*, *E. hormaechei* and *E. cloacae* (Fig. 5G). Furthermore, the growth curves of the five bacteria showed that Defensin1 significantly suppressed the growth of *S. alvi*, *L. kullabergensis,* and *L. helsingborgensis* (*P* < 0.01), whereas Hymenoptaecin significantly inhibited the growth of *L. kullabergensis* and *L. helsingborgensis* (*P* < 0.01) (Fig. 5H)*.* The results indicated that CBPV infection caused an excess of AMPs, which in turn decreased the abundance of the core gut probiotics species and further promoted the abundance of opportunistic pathogenic bacteria, such as *E. hormaechei* and *E. cloacae,* to facilitate viral infection.

## Discussion

**Figure 6:**
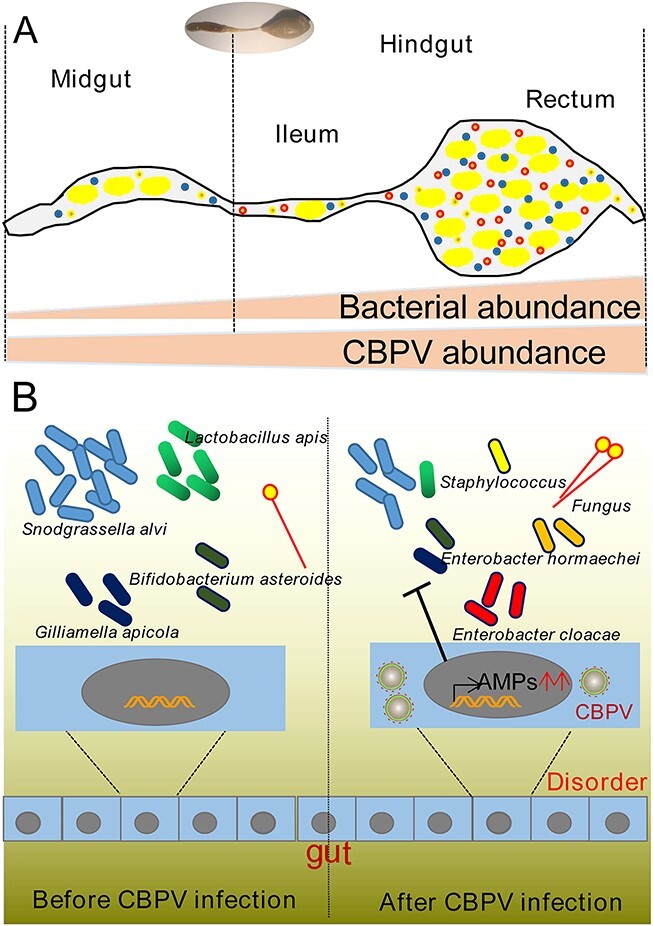
A model for gut immune modulation and CBPV infection amplification by the hindgut microbiota has been proposed. (A) A diagram of the bee gut, showing the midgut and hindgut. The regions of the hindgut known as the ileum and rectum exhibit greatest bacteria densities and abundance of CBPV. (B) CBPV infection induces AMPs expression to change gut microbiota composition, including decreasing the abundance of probiotic species (such as *S. alvi, L. apis*, *G. apicola*, and *B. asteroides*) and increasing the abundance of opportunistic bacteria (*E. cloacae*, *E. hormaechei*, *Staphylococcus* spp.).

Since its identification in 1963 [10], CBPV has spread across bee colonies worldwide, resulting in significant manifestations such as bloated abdomen. While existing research has primarily concentrated on the prevalence and occurrence of CBPV in both wild and managed honey bee populations by PCR, there remains a dearth of understanding regarding the mechanisms by which the virus evades the host’s immune response and induces characteristic symptoms [11, 46, 47]. Our study revealed that the CBPV proliferation exhibited the highest titers of the virus and resulted in the symptoms of bloated abdomen. Thus, we propose a model in which the CBPV utilizes the host immune pathway that produces excess AMPs to reduce the abundance of core probiotics species in the gut and then promote the abundance of opportunistic pathogenic bacteria to facilitate viral infection (Fig. 6).

Most bacterial species clusters are dominated in the hindgut of honey bee workers [38]. Our results showed that CBPV was distributed mainly in the hindgut, followed by the brain, midgut, thorax, fat body, and trachea, and that its infection resulted in more cell degradation in the hindgut (Fig. 1). It has been reported that the prevalence of honey bee viruses in queens showed that the head was not the primary tissue for CBPV infection [46]. Our previous investigation suggested that the replication level of the head and thorax was significantly higher than that of the abdomen when orally infected [11]. These findings indicated that regardless of the route of infection, CBPV can cause systemic infection in major tissue such as the hindgut and head. Physical barriers, cell-mediated immunity, and humoral immunity are three levels of indirect immunological control employed by honey bees to combat gastrointestinal pathogens [48]. Our results indicated that the immune genes related to the Toll/Imd pathway were considerably up-regulated in *A. mellifera* after CBPV infection (Fig. 4, Table S7). Furthermore, AMPs, such as *hymenoptaecin* and *defensin1* were significantly up-regulated at least more than 100 times after CBPV infection, which were always at least 10 times higher than those of IAPV-infected *A. mellifera* during the survey period [8] (Fig. 4). Previous reports revealed that the levels of DWV or BQCV infection were positively correlated with the expression of *hymenoptaecin* and *defensin1* [49]. In fact, our results showed that CBPV significantly induced the expression of AMPs at Days 1, 2, 3, and 4, but the CBPV titer was still higher at hindgut and midgut at Day 4 (Figs 1 and 4). AMPs have been shown to play a crucial role in eliminating pathogens and maintaining the equilibrium of gut microbiota [19, 20]. These findings have prompted the hypothesis that AMPs such as *hymenoptaecin* and *defensin1* may uphold a specific level of expression in honey bees to regulate the composition of gut microbiota during viral infection.

The AMPs are closely associated with bee humoral immune response and exhibit broad antibacterial activities against gram-positive and gram-negative bacteria [50]. Consistent with those, our study showed that tetracycline treatment significantly reduced the abundances of core species *G. apicola*, *S. alvi*, and *L. apis* (Fig. 3), and then the expression levels of most AMPs was significantly decreased, such as *hymenoptaecin* and *apisimin* (Fig. S6). Our results also showed that CBPV infection significantly induced the expression of AMPs and decreased the relative abundances of the probiotic bacteria *S. alvi*, *L. apis*, *G. apicola*, and *B. asteroides* (Figs 2 and 4). Spearman correlation analysis showed that the expression levels of *defensin1* and *hymenoptaecin* induced by CBPV infection had a strong negative correlation with the abundances of core probiotics, such as *Snodgrassella* and *Lactobacillus* (Fig. 5). Likewise, a recent study showed that AMPs are negatively correlated with the relative abundance and density of core species in *Bactrocera dorsalis* [25]. As expected, Defensin1 had slight antibacterial activity on Gram-negative *S. alvi*, and definite antibacterial activity on Gram-positive *L. kullabergensis* and *L. helsingborgensis*, and hymenoptaecin had strong antibacterial activity on Gram-positive *L. kullabergensis* and *L. helsingborgensis.* Consistent with the latter, AMPs regulate gut microbiota composition and abundance of core species in *Drosophila* [45]. Specifically, it has been verified that the presence of *S. alvi*, *L. apis,* and *G. apicola* leads to the formation of a biofilm on the gut ileum wall, serving as a physical barrier against pathogen invasion [3]. *S. alvi* recognized as a probiotic in both humans and other animals, including honey bees [51], whereas *G. apicola* has been identified as a probiotic with potential efficacy against pathogen infiltration in animals [52]. In vivo experimental evidences showed that *S. alvi* and *G. apicola* induced honey bee Toll/Imd pathway in response to *Escherichia coli* infection [39]. Combined with the reported literature that AMPs targeted symbiotic bacteria [25, 45], we speculated that overabundance of AMPs induced by CBPV may specifically target core bacteria, potentially leading to a significant alteration in the typical composition of the gut microbiota in honey bees.

CBPV infection in honey bees was also distinguished by a relatively high proportion of *E. hormaechei* and *E. cloacae* in *A. mellifera* (Fig. 2). It has been reported that a majority of enterobacteria were sensitive to tetracycline [44], and the tetracycline treatment can effectively reduce the proliferation of CBPV in honey bees and alleviated the typical symptoms of bloated abdomens in CBPV-infected bees (Fig. 3). Associated studies have shown that antibiotic-treated mice were less susceptible to poliovirus (enteric virus) disease and sustained low viral replication in the intestine [44, 53]. Gut microbiota can enhance proliferation and transmission of multiple enteric RNA viruses through a variety of ways in mammalian [35, 54]. The microbiota, for example, can suppress innate immune responses, increase viral infectivity by assisting viral attachment to host cells, or improve virion stability [35]. In our study, spearman correlation analysis showed that the expression levels of *defensin1* and *hymenoptaecin* induced by CBPV infection had a strong positive correlation with both of them (Fig. 5). Furthermore, Defensin1 and Hymenoptaecin had no obvious antibacterial activity on *E. hormaechei* and *E. cloacae* (Fig. 5) [55]. Some studies have suggested that AMPs have spectral activity that can protect against fungal and bacterial infections by interacting with the membranes or acting as cell wall inhibitors [56, 57]. However, a recent study found that the AMPs cecropin 3 or lebocin fail to suppress the proliferation of the entomopathogenic fungus *Metarhizium rileyi* [58]. *E. cloacae* complex strains are intrinsically resistant to a number of antibiotics (including carbapenem and colistin) and have demonstrated a remarkable ability to acquire additional resistance determinants, including beta-lactamases and extended spectrum carbapenemases, which in some cases represent the available antibiotics treatment significantly limit antibiotic treatment [59], even AMPs treatment, suggesting that the presence of antibiotic resistance genes within these bacterial strains may precipitate an ecological crisis. This is due to the potential for vertical transfer of these genes to subsequent generations of bacteria, leading to a proliferation of resistant strains, and then alters to the microbial composition and community dynamics within the ecosystem, thereby disrupting its equilibrium. Additionally, the horizontal transfer of these genes to other organisms and their subsequent integration into the food chain poses a significant threat to human public health.

The significant difference analysis of gut microbiome function between CBPV-infected and healthy honey bees using multiple t-tests indicated that cell motility was positive related to CBPV infection (Fig. S5). Likewise, it has been reported that chronic hepatitis B virus (HBV) infection can reduce the numbers of intestinal *Lactobacillus* and increase the absolute abundance of *Enterobacteriaceae,* which are linked with the loss of intestinal microvilli, the widening of the intestinal mucosal space and intestinal bacterial translocation [29, 60]. Furthermore, enteric viruses can bind to bacteria via polysaccharides on their surfaces [35]. Our results showed that CAZymes function annotation analysis revealed that genes encoding carbohydrate esterases were significantly clustered in CBPV infection group (Fig. S5), suggesting that CBPV may also bind to bacteria via bacterial surface polysaccharides, allowing for increased proliferation. Moreover, our study also showed that the treatment of *E. hormaechei* and *E. cloacae* slightly promoted the viral proliferation, and obviously worsened the typical symptoms of bloated abdomens in CBPV-infected bees. This treatment also led to the disruption and thinning of gut microvilli in CBPV-infected honey bees (Fig. 3). Microvilli are known to be involved in food digestion, nutrient absorption, and acting as a physical barrier against pathogens in insects [61]. Considering that the bacteria of the *E. cloacae* complex adhere to and invade epithelial cells and induce apoptosis including nuclear chromatin, formation of apoptotic bodies and cell membrane blebbing [62], this could be the reason that CBPV infection promoted the *E. hormaechei* and *E. cloacae* proliferation, and then the epithelial cells of the hindgut became shed and thin from day 4, and thereby resulted in difficulty of defecating and forming typical symptom of the bloated abdomen after CBPV invasion in honey bees.

Overall, our findings suggested the Toll/Imd pathway was activated to produce more AMPs, thereby influencing significant changes in the composition of gut microbiota to facilitate CBPV infection in honey bees (Fig. 6). These findings added to our understanding of the different mechanism of viral infection: CBPV utilizes the host immune system to diminish the population of probiotic core species, and then boosts the opportunistic pathogens proliferation for facilitating viral infection.

## Supplementary Material

supplementary_information3_wrae051

Supplementary_Table_S4-S8_wrae051

## Data Availability

The raw reads of metagenomic sequencing datasets were deposited at the public database Genome Sequence Archive (http://gsa.big.ac.cn/) under the accession number CRA006394.
