# Supplementary material for: Chronic bee paralysis virus exploits host antimicrobial peptides and alters gut microbiota composition to facilitate viral infection
Source: ISME J. 2024 Mar 22;18(1):wrae051. doi: 10.1093/ismejo/wrae051 (PMC11014883; doi:10.1093/ismejo/wrae051)
Supplement: supplementary_information3_wrae051 [file supplementary_information3_wrae051.docx]

**Supporting Information for**

**Chronic bee paralysis virus exploits host AMPs and alters gut microbiota composition to facilitate viral infection**

**Running title: CBPV altered the host microbiota composition to maintain viral infection**

**Yanchun Deng ^1,#^, Sa Yang ^2,3,#^, Li Zhang^2,#^, Chenxiao Chen^1^, Xuefen Cheng ^1^, Chunsheng Hou ^1,*^**

^1^ Institute of Bast Fiber Crops, Chinese Academy of Agricultural Sciences, Changsha 410205, People’s Republic of China

^2^ Institute of Apicultural Research, Chinese Academy of Agricultural Sciences, Beijing 100193, People’s Republic of China

^3^ Graduate School of Chinese Academy of Agricultural Sciences, Beijing 100081, People’s Republic of China

^#^ These authors contributed equally to this work. Author order was determined on the basis their contribution.

**^*^** Corresponding author: [houchunsheng@caas.cn](mailto:houchunsheng@caas.cn)

**MATERIALS AND METHODS**

**Honey bee**

Three adult worker honey bee (*A. mellifera*) colonies were collected from Guangdong Institute of Applied Biological Resource, Guangzhou, China. The six brood frames from the three different colonies were transferred into an incubator (30 ± 1℃, 60% relative humidity (RH), and newly emerged honey bees within 24 hours were collected for the following experiments. These honey bees were seemingly identified as healthy, and were free from bacterial diseases (American foulbrood, European foulbrood) with the special primers (**Table S1**), according to a previously published method [1]. They were also free from *Varroa* mites and fungal diseases (*Nosema*, Chalkbrood and Stonebrood) under a microscope. Simultaneously, these honey bees were screened for the presence of common honey bee viruses, including IAPV, SBV, DWVa, DWVc, CBPV, Chinese sacbrood virus (CSBV), BQCV, KaKugo virus (KV), aphid lethal paralysis virus (ALPV) and ABPV with the special primers (**Table S1**) by conventional PCR, as previously described [2]. After that, about 30 newly emerged honey bees were transferred into one standard wooden cage (8 cm × 6 cm × 12 cm) as one repeat (three repeats for each group, 90 honey bees per group), and all the cages were kept in an artificial climate incubator (MGC-800HP, shanghai, China) with 2 mL 50% sucrose syrup provided every day.

**Infection on honey bees with CBPV**

Since an accurate characterization of the interactive relationship between viruses and the host is not possible due to the lack of CBPV cell culture systems *in vitro*, the construction of infectious clones has become a feasible way to study the pathogenesis of honeybee viruses, as our previous study led to the construction of one CBPV infectious clone [3]. Briefly, the RNA1 and RNA2 fragments of CBPV were amplified using RT-PCR with the high-fidelity Phusion HiFi PCR Master Mix (NEB, Ipswich, MA, USA). The full length infectious clone (pACYC177-CBPV-RNA1 and pACYC177-CBPV-RNA2) were completed by ClonExpressMultiS One-Step Cloning Kit (Vazyme Biotech Co., Nanjing, China) with the low copy vector pACYC177 (CWBio, Beijing, China) according to manufacturer’s instructions. For in vitro RNA synthesis, firstly, we used a Plasmid MiniPrep Kit (TransGen Biotech, Beijing, China) to extract each stable plasmid pACYC177-CBPV RNA1 and pACYC177-CBPV RNA2, which was used as the template to amplify the full-length IAPV by high-fidelity Phusion HiFi PCR Master Mix (NEB, Ipswich, MA, USA). Subsequently, we used Dpn I and a QIA Quick gel extraction kit (Qiagen, Hamburg, Germany) to treat and recycle PCR production. Finally, we used a HiScribe T7 Quick High Yield RNA Synthesis Kit (NEB, Ipswich, MA, USA) to finish the in vitro transcription reaction by utilizing the recycled PCR production according to the manufacturer’s recommended protocol, and the RNA was purified with an EasyPure RNA Kit (TransGen Biotech, Beijing, China).

These honey bees were injected with 2 µL of purified synthetic CBPV RNA1 and RNA2 (approximately 1×10^12^ genome copies) into the third to the fourth integument of the honey bee abdomen with a Hamilton syringe (702) (Hamilton, Switzerland), respectively (**Fig. 1A**). The control group was injected with PBS, and the process of injecting all honey bees with CBPV RNA or PBS was completed in two hours. After injection, these groups were transferred to an incubator at 30℃/60% RH, and dead honey bees were observed and recorded daily.

**Tissue dissection and histopathological examination**

To study the damage caused by CBPV infection in the gut, histopathological analysis of the midgut and hindgut of honey bees was performed as described previously [4]. Briefly, these tissues were taken from five honey bees at days 4, 6, and 8 after CBPV infection. These honey bees were from each of the three colonies maintained in an incubator and then quickly fixed with 4% paraformaldehyde and stored at 4℃ for 12 hours. Next, we performed conventional dehydration and paraffin embedding, and then the slides with tissues were dewaxed in dimethyl benzene and rehydrated in alcohol of diminishing concentrations. Subsequently, hematoxylin and eosin (HE) staining were finished to examine the pathological changes in the midgut and hindgut under a Leica DFC280 light microscope and analyzed using the Leica Q Win Plus V3 Image Analysis System (Leica Micros Imaging Solutions Ltd.; Cambridge, UK).

**DNA extraction and metagenome sequencing**

The entire digestive tract of five honey bee including malpighian tubules but excluding the crop, was carefully collected from each honey bee using sterile forceps as previously described [5]. These gut samples were then transferred into a new centrifuge tube and stored at −80 ℃. Genomic DNA of gut microbial from 10 PBS-injected and CBPV-infected honey bees was extracted from frozen gut using a total DNA extraction kit provided by Hangzhou Foreal Nanotechnology (Hangzhou, China) according to the manufacturer's instructions. The extracted DNA was quantified using a Qubit 3 Fluorometer (Invitrogen, USA) and stored at −80°C for further analysis. The integrity and purity of the extracted DNA were checked through agarose gel electrophoresis.

The microbial genomic DNA obtained was subjected to sequencing, and the raw data generated from a HiSeq System (Illumina) was processed using Readfq (V8, <https://github.com/cjfields/readfq)> to obtain clean data for subsequent analysis at Novogene (Beijing, China). Statistical analysis of the sequence data was performed. In brief, by SOAPdenovo software (V2.04, <http://soap.genomics.org.cn/soapdenovo.html)>, the clean data was assembled and analyzed and the parameters were as in previous studies [6-8]. Then all samples’ clean data are compared to each scaffold by Bowtie2.2.4 software to acquire the PE reads, respectively [6]. After that, DIAMOND software (V0.9.9, https://github.com/bbuchfink/diamond/) was used for blasting the Unigenes to the sequences of Bacteria, Fungi, Archaea and Viruses, which were all extracted from the NR database (Version: 2018-01-02, https://www.ncbi.nlm.nih.gov/) of NCBI. As each sequence may have multiple aligned results, choose the appropriate result to take the LCA algorithm which was applied to the system classification of MEGAN software to make sure the species annotation information of sequences to get the finally aligned results of each sequence [9-10]. In view of the LCA annotation result and the gene abundance table, we obtain the table including the number of genes and the abundance information of each sample in each taxonomy hierarchy (kingdom, phylum, class, order, family, genus, species). Then Metastats and LEfSe analysis were used to look for the various species between groups and a permutation test between groups was used in Metastats analysis for each taxonomy and get the P-value. After that, we used Benjamini and Hochberg False Discovery Rate to correct P-value and acquire the q value [11]. The principal component analysis (PCA) was performed to investigate whether gut bacteria clustered at the basic role level by the R (v4.0.3) packages (psych, reshape2, ggplot2 and factoextra). Next, we adopted DIAMOND software (V0.9.9) to blast Unigenes to the functional database, KEGG database (Version 2018-01-01, <http://www.kegg.jp/kegg/),> and for the blast result of each sequence, the best Blast Hit was used for subsequent analysis [12]. Carbohydrate-Active enzymes Database (CAZy) database was used as a professional database for the study of carbohydrate enzymes, mainly contains 6 functional categories: Glycoside Hydrolases (GHs), Glycosyl Transferases (GTs), Polysaccharide Lyases (Polysaccharide Lyases, PLs), Carbohydrate Esterases (CEs), Auxiliary Activities (AAs) and Carbohydrate-Binding Modules (CBMs) [13].

**RNA extraction and Quantitative Real-Time PCR (qPCR) analysis**

Total RNA was extracted and the cDNA synthesis was performed with 1000 ng of RNA using the PrimeScript RT Reagent Kit with gDNA Eraser (TaKaRa, Dalian, China) according to the manufacturer’s instructions.

Quantification of the replication level of CBPV was performed using qPCR with the specific primers (**Table S2**). qPCR analysis was performed using TB Green Premix Ex Taq II (Takara, Dalian, China) at Applied Biosystems 9600 Real-Time PCR system (Hangzhou, China). The specificity of the primers was confirmed by the melt curve analysis. Viral loads were quantified by absolute quantification methods. Briefly, the linear standard curve equation for the virus was based on the standard linear curve obtained through six ten-fold dilutions of known amounts of plasmids (pMD 18-T Vector, TaKaRa) containing cloned viral target sequences, and the standard linear curve was used for calculating the copies and the standard linear curves of viral and immune genes were obtained in this study (**Table S3)**. The qPCR for immune genes and AMPs was performed using the specific primers (**Table S2)**, and the general protocol was similar to that of the CBPV.

**Analysis of the expression of immune genes by RNA-seq**

The extracted RNA of each sample of PBS and CBPV-injected adult honey bees was used for library construction and RNA-seq at Novogene (Beijing, China). Briefly, the enriched mRNA was fragmented into approximately 200-nt RNA inserts to synthesize the double-stranded cDNA. Then the end-repair/dA-tail and adaptor ligation were finished. Subsequently, the corresponding fragments were obtained with Agencourt AMPure XP beads (Beckman Coulter, Inc., USA) and then by PCR amplification using Phusion High-Fidelity DNA polymerase, universal PCR primers and Index (X) Primers. PCR products were purified (AMPure XP system) and the quality of the library was assessed with the Agilent Bioanalyzer 2100 system. After that, the clustering of the index-coded samples was finished by the cBot Cluster Generation System using TruSeq PE Cluster Kitv4-cBot-HS (Illumina, Inc., United States) following the method of instructions and the library preparations were sequenced on an Illumina Hiseq 2500 platform and single-end reads were generated. Next, the house Perl scripts were used to analyze the raw data in FASTQ format, and clean reads were obtained by removing those reads containing adapter, ploy-N, and low-quality. The clean reads filtered from the raw reads were mapped to the *Apis mellifera* genome (Aml-4.5) using Tophat2 software [14]. The cufflinks software was used TO estimate gene expression levels using FPKM values (fragments per kilobase of exon per million mapped fragments value) [15]. To get a better analysis of differential gene expression analysis in immune response, weighted gene co-expression network analysis (WGCNA) was used. WGCNA, which belongs to the R package, is an algorithm for excavating module information based on the gene chip expression data [16]. The similar expression patterns of different genes are considered to be in the same co-expression network or module, and the common relationship between genes is determined by the correlation coefficient [16]. The interaction networks of corresponding proteins of the target genes were predicted using STRING (https://cn.string-db.org/).

**Treatment CBPV-infected honey bees with tetracycline**

To eliminate the majority of gut bacterial populations, the tetracycline dosage was adopted according to the previously published article [17]. Newly emerged honey bees were randomly assigned to receive tetracycline at a concentration of 500 μg/mL (equivalent to the median lethal doses for honey bees, LD50), whereas a sterilized 50% sugar solution was supplied as a control. After 2 days, the honey bees were subjected to RNA injection of CBPV and were continuously fed with tetracycline for 8 days. Daily observations of mortality were conducted. Then, five honey bees from each group were collected to assess CBPV proliferation levels at days 1, 3, 5, and 7 post-CBPV infection. All other experimental conditions remained consistent with those detailed in the preceding sections. The samples following CBPV infection with tetracycline treatment were photographed using reflected-light stereomicroscope (Leica, Germany).

To detect the specific bacterial abundance after tetracycline treatment in CBPV-infected honey bees, microbial genomic DNA was extracted from the frozen feces of five bees using E.Z.N.A. Stool DNA Kit (Omega, America) following the manufacturer’s instructions. The extracted microbial genomic DNA was used to quantify the relative abundance of bacteria by qPCR according to a previously published method [18], with specific bacterial phyla primers in previous studies [19, 20].

**The opportunistic pathogens treatment in CBPV-infected honey bees**

Various strains of gut bacteria from honey bee, including *S. alvi*, *Lactobacillus kullabergensis*, *Lactobacillus helsingborgensis*, *E. cloacae*, and *E. hormaechei,* were obtained from CBPV-infected-bees (*A. mellifera*). The bacteria were isolated using Luria-Bertani (LB) and de Man Rogosa Sharpe (MRS) broths (**Fig. S10**). The identification of the isolated strains was determined through PCR analysis using the 16S rRNA genes, as described in a previous study [21]. Then, a single isolated colony of each strain was cultured in liquid broth, with *S. alvi* incubated in LB broth under static conditions at 37℃, and *L. kullabergensis*, *L. helsingborgensis*, *E. cloacae*, and *E. hormaechei* incubated in MRS broth under static condition at 37℃. The broths were cultured overnight and their optical density at 600 nm (OD_600_) was determined using a spectrophotometer (UV-2700, Shimadzu, China).

The study investigated the influence of the gut microbiota on viral proliferation by conducting an experiment on the treatment of opportunistic pathogen. For this purpose, 10 mL culture broth containing *E. cloacae* and *E. hormaechei* (OD_600_ value = 0.6) were centrifuged at 8000 rpm and 37℃ for 60 s. The resulting precipitate was then suspended in a 10 mL 50 % sucrose solution, and the two contaminated sugar solutions were mixed for further analysis. Newly emerged honey bees were randomly divided into groups to be fed the mixture of the *E. cloacae*, *E. hormaechei* and sucrose syrup, with a sterilized 50% sugar solution supplied as a blank control. After one day, the honey bees were injected with CBPV and continuously fed with the contaminated mixture for 7 days, with daily observation of mortality. Five honey bees from each group were collected on days 1, 3, 5 and, 7 after CBPV infection to determine the levels of CBPV proliferation. The experimental conditions remained consistent with those described in the preceding sections. A reflected light stereomicroscope (Leica, Germany) was used to record the samples following CBPV infection with opportunistic pathogen treatment.

**Expression and purification of AMPs proteins, and analysis of the antibacterial activity of AMPs**

To obtain soluble Defensin1 and Hymenoptaecin proteins *in vitro*, plasmids of pET28a-Defensin1 and pET28a-Hymenoptaecin were constructed according to the manufacturer’s manuals (**Table S1**). Briefly, the above cDNA was used to amplify full-length of *defensin1* and *hymenoptaecin* with the high-fidelity Phusion HiFi PCR Master Mix (NEB, Ipswich, MA, USA) using specific primers.

For purification of the fusion proteins by His Pur Ni-NTA Resin (Thermo Scientific, Beijing, USA), the 250 mL of IPTG-induced bacterial solution was centrifuged at 4℃ and 12000 rpm for 10 minutes, and the pellet was resuspended in 25 mL of 1X PBS (pH 7.4). About 25 mL of the filtrated supernatant contained targeted soluble protein was loaded onto the Gravity column with 1 mL of His Pur Ni-NTA Resin, and then the protein was eluted with 5 mL of different concentrations of imidazole (25, 50, 250, and 500 mM in PBS buffer). Low concentrations of imidazole washed unbound various proteins and the purified protein was collected in 5 mL of 500 mM imidazole and concentrated through ultrafiltration tube, followed by dialysis in PBS buffer.

The Oxford cup method was used to determine the inhibitory activity of AMPs induced by CBPV infection against core probiotic species and opportunistic pathogens. The above strains of *S. alvi*, *L. kullabergensis*, *L. helsingborgensis*, *E. cloacae*, and *E. hormaechei* were cultured with respective broth until the OD_600_ value became 0.6, then 0.5 ml of the 5 bacterial suspensions was spotted onto the LB or MRS plates, respectively. Nine Oxford cups for three groups were placed 1 cm apart on the surface of the plate, and then 0.1 mL of specified concentration (0.5 mg/mL) of the purified protein (Defensin or Hymenoptaecin) and BSA (control) were added to the Oxford cup and cultured at 37℃ for 24 hours. The diameter of the zone of inhibition was observed and the tests were repeated three times.

To further confirm the inhibitory activity of Defensin1 or Hymenoptaecin (0.02 mg/mL) against the gut microbiota, the growth curves were performed. *S. alvi*, *L. kullabergensis*, and *L. helsingborgensis* were incubated in MRS broth under static condition at 37℃, whereas *E. cloacae*, and *E. hormaechei* were incubated in LB broth at 200 rpm and 37℃. The optical density at 600 nm (OD600) of the overnight cultured broths was determined using a spectrophotometer (UV-2700, Shimadzu, China) to assess their growth.

**Supplementary figures**


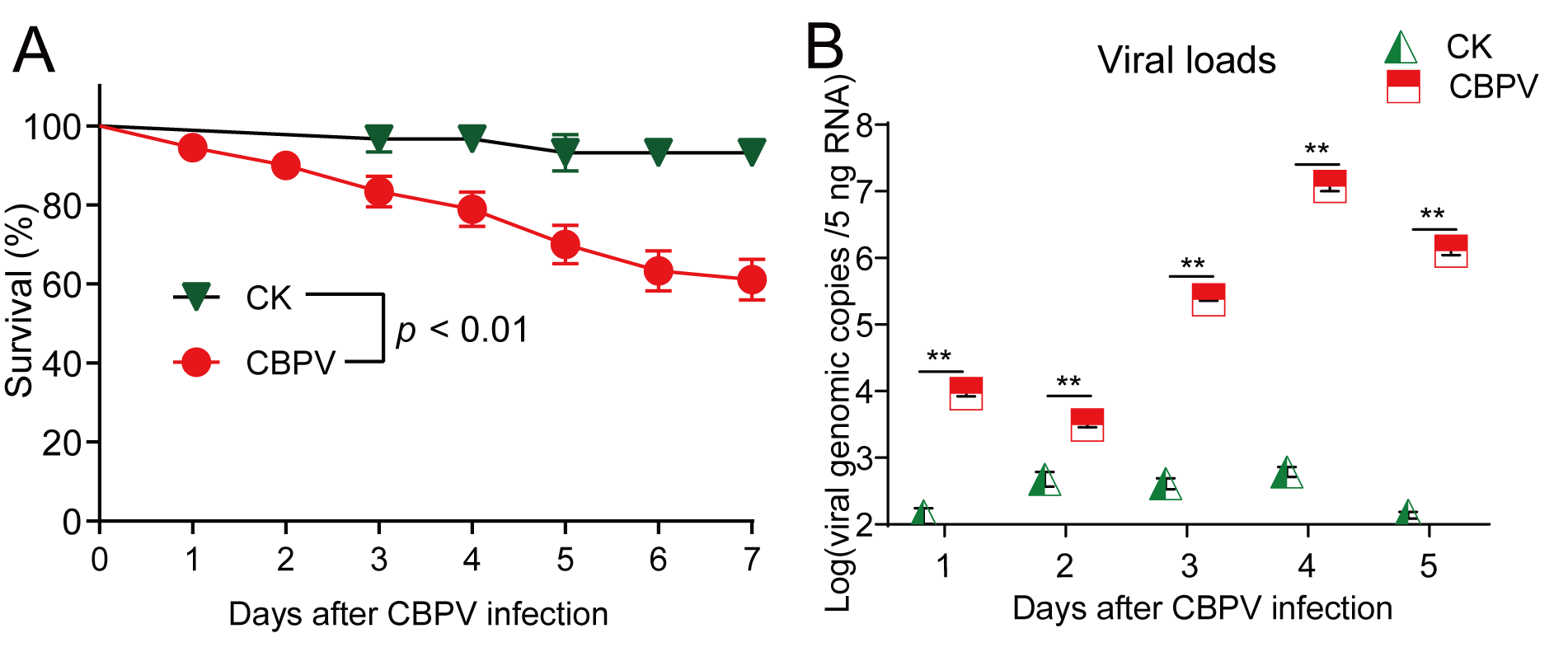


**Fig. S1 The survival rate and viral proliferation in newly emerging *Apis mellifera* infected with CBPV.** (A) Survival rates of the newly emerging honey bees infected with and without CBPV infection. (B) The viral proliferation of the newly emerging honey bees infected with CBPV infection.


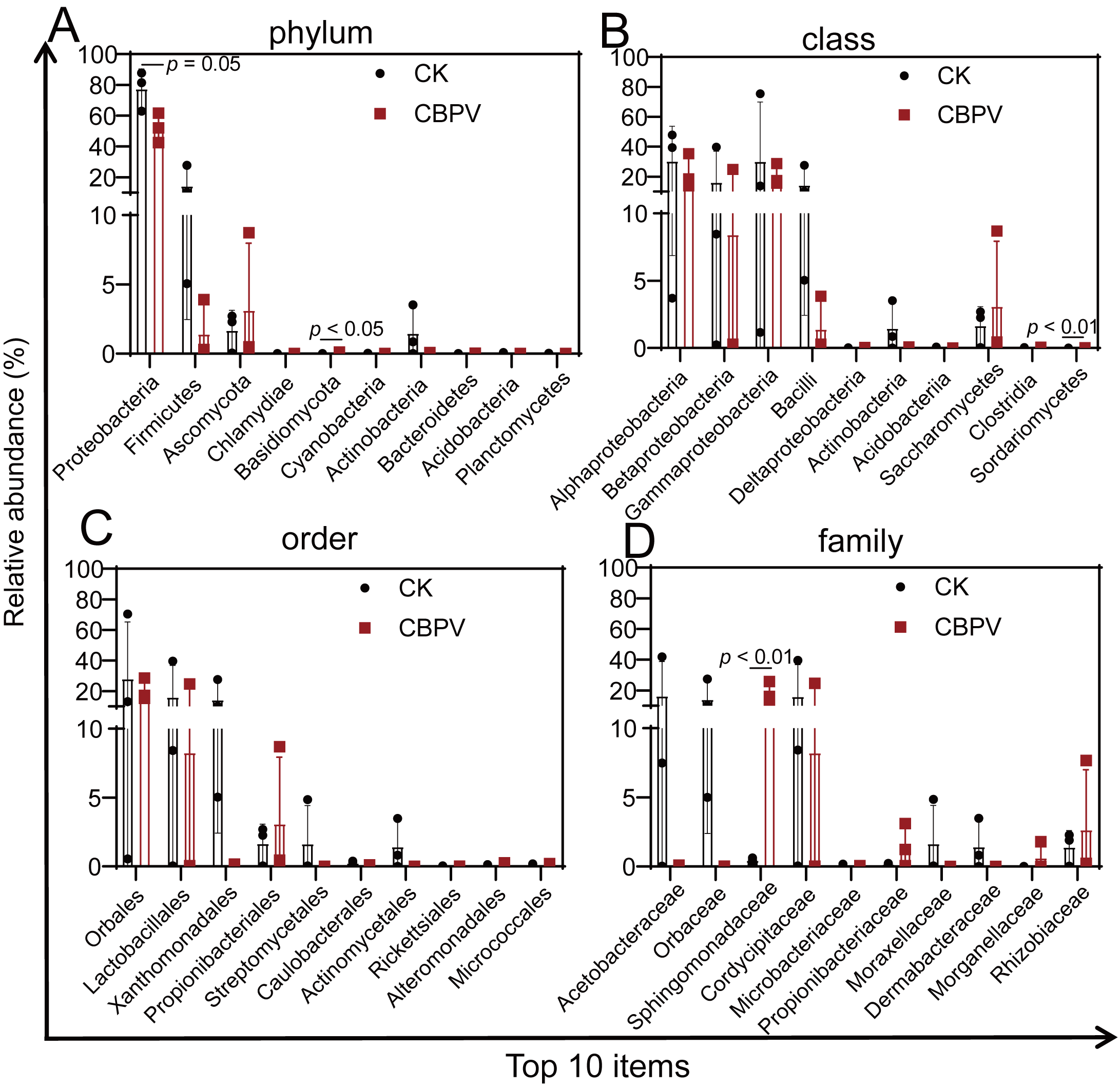


**Fig. S2 Viral infection affects the bacterial community in *A. mellifera* at days 5.** The column graph showed the relative abundances of gut bacterial species in healthy and CBPV-infected honey bees at phylum (A), class (B), order (C) and family (D) level.

**
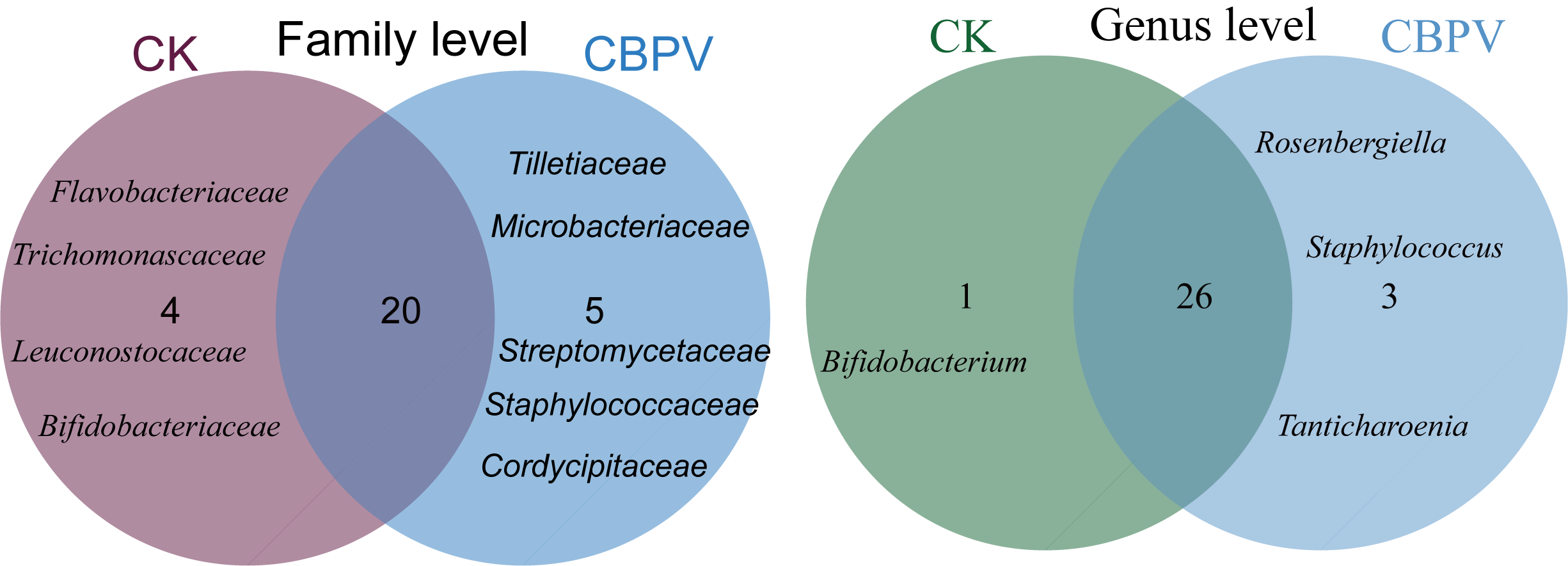
**

**Fig. S3 Viral infection affects the bacterial community in *A. mellifera* at days 5.** Venn diagrams showing shared and unique taxa at the family and genus level (relative abundance >0.01%) for the control and CBPV-infected groups.


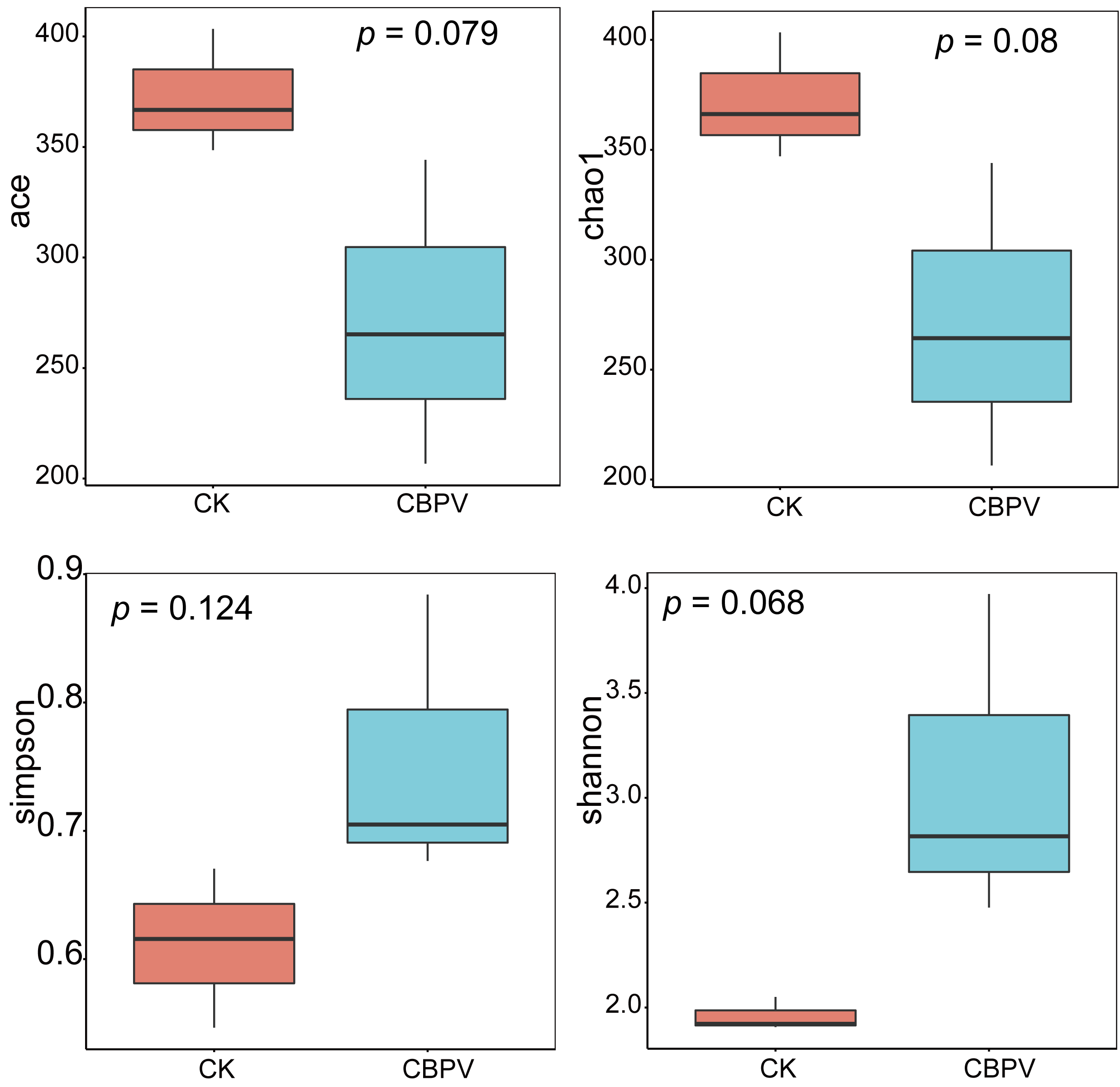


**Fig. S4 Alpha diversity analysis after CBPV infection in *A. mellifera*.** (A) ace index. (B) chao 1 index. (C) simpson index. (D) shannon index.

**
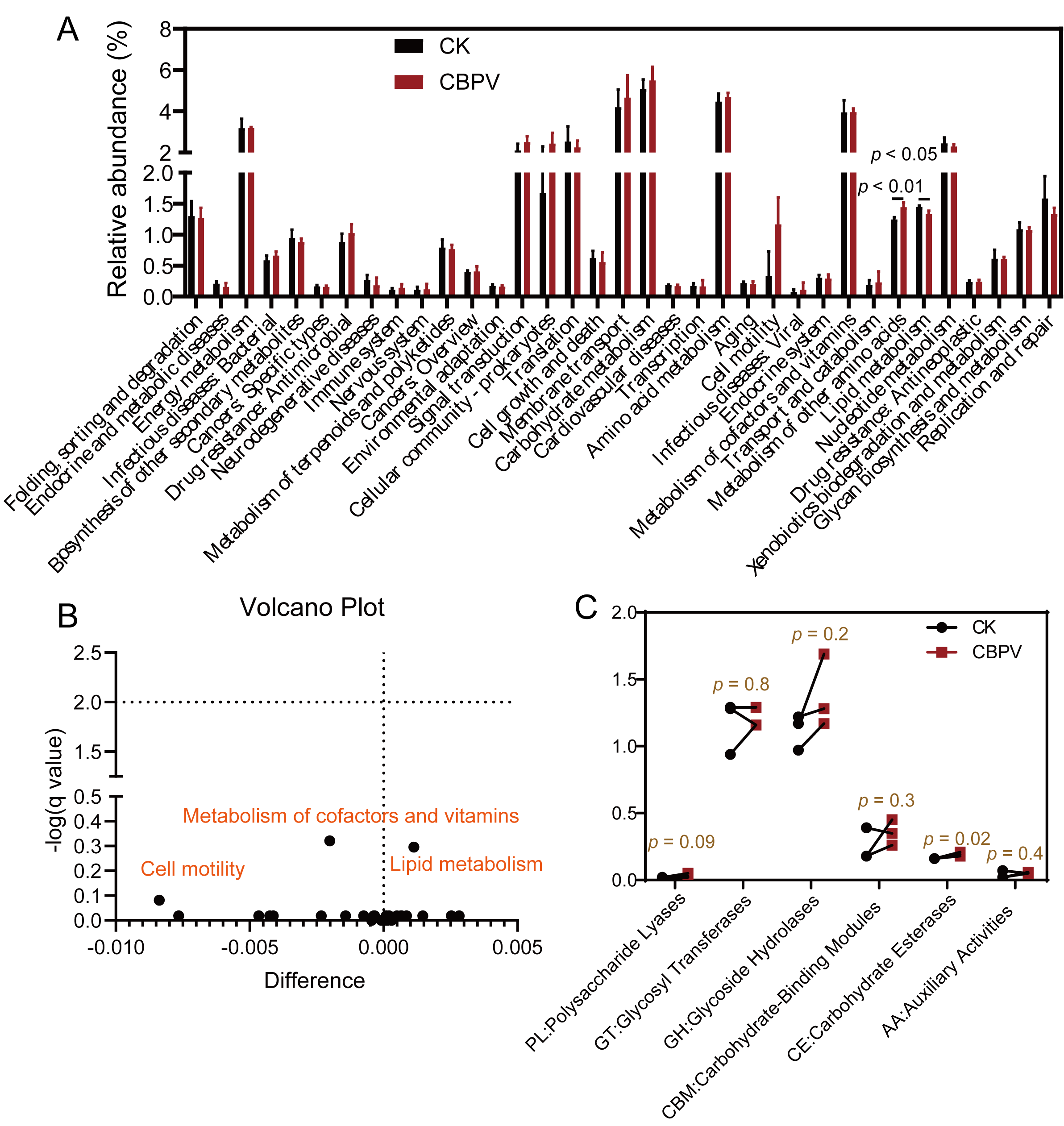
**

**Fig. S5 Viral infection affects the functional pathway of microbiota in CBPV-infected-bees at days 5.** (A) The column graph showed the absolute abundances of gut microbiota related to different KEGG modules in healthy and CBPV-infected honey bees. (B) The well represented KEGG pathway found in the gut microbiota of honey bees infected with CBPV (relative abundance > 1%). (C) The highly represented CAZyme family functional annotations found in the gut microbiome of bees infected with CBPV (relative abundance > 1%).


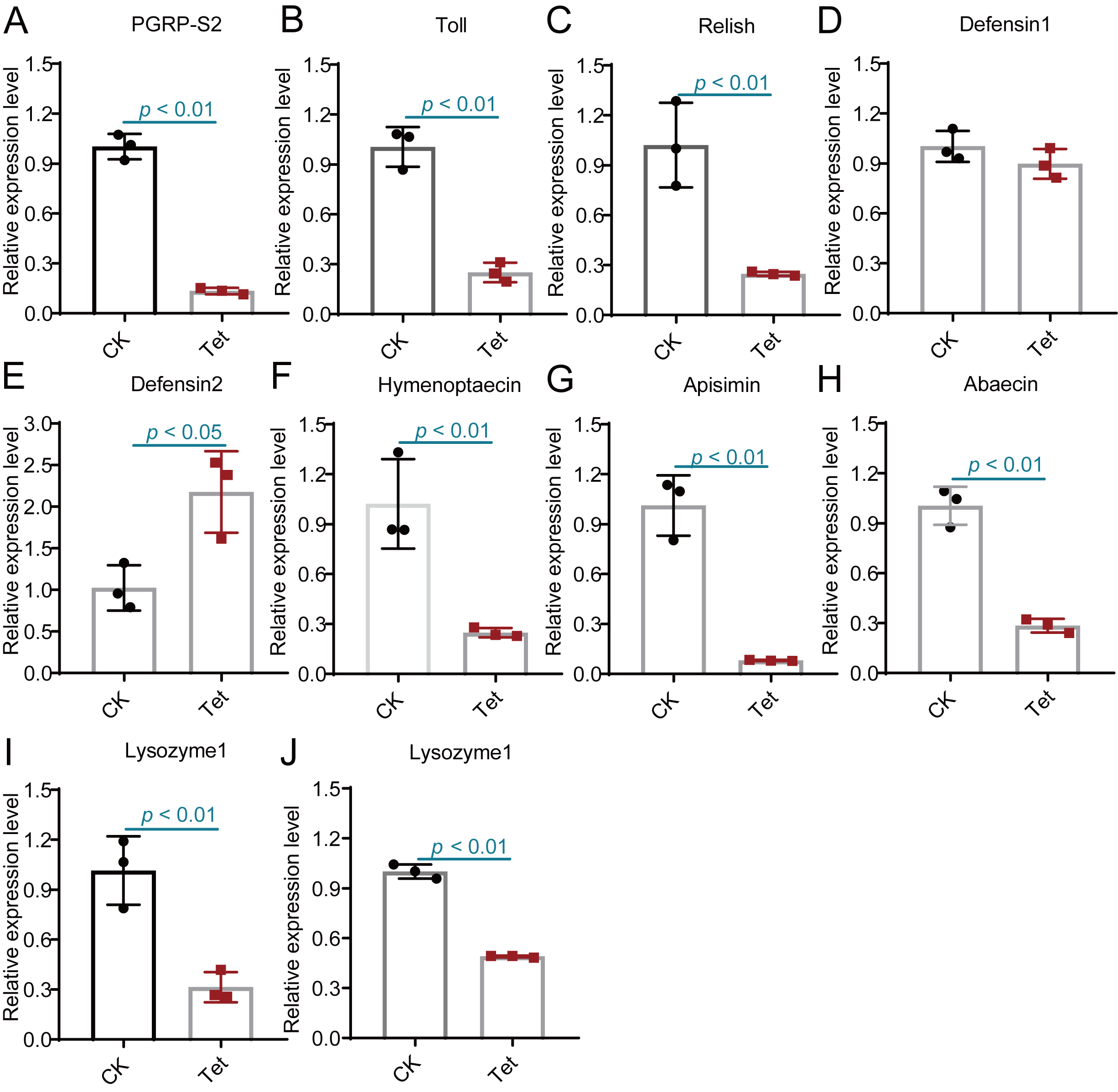


**Fig. S6 The comparative analysis of expression level of Toll/Imd pathway and antimicrobial peptide genes in newly emerging *A. mellifera* treated with tetracycline at days 5.** (A-C) The expression levels of genes in Toll/Imd pathway. (D-J) The expression levels of AMPs genes.

**
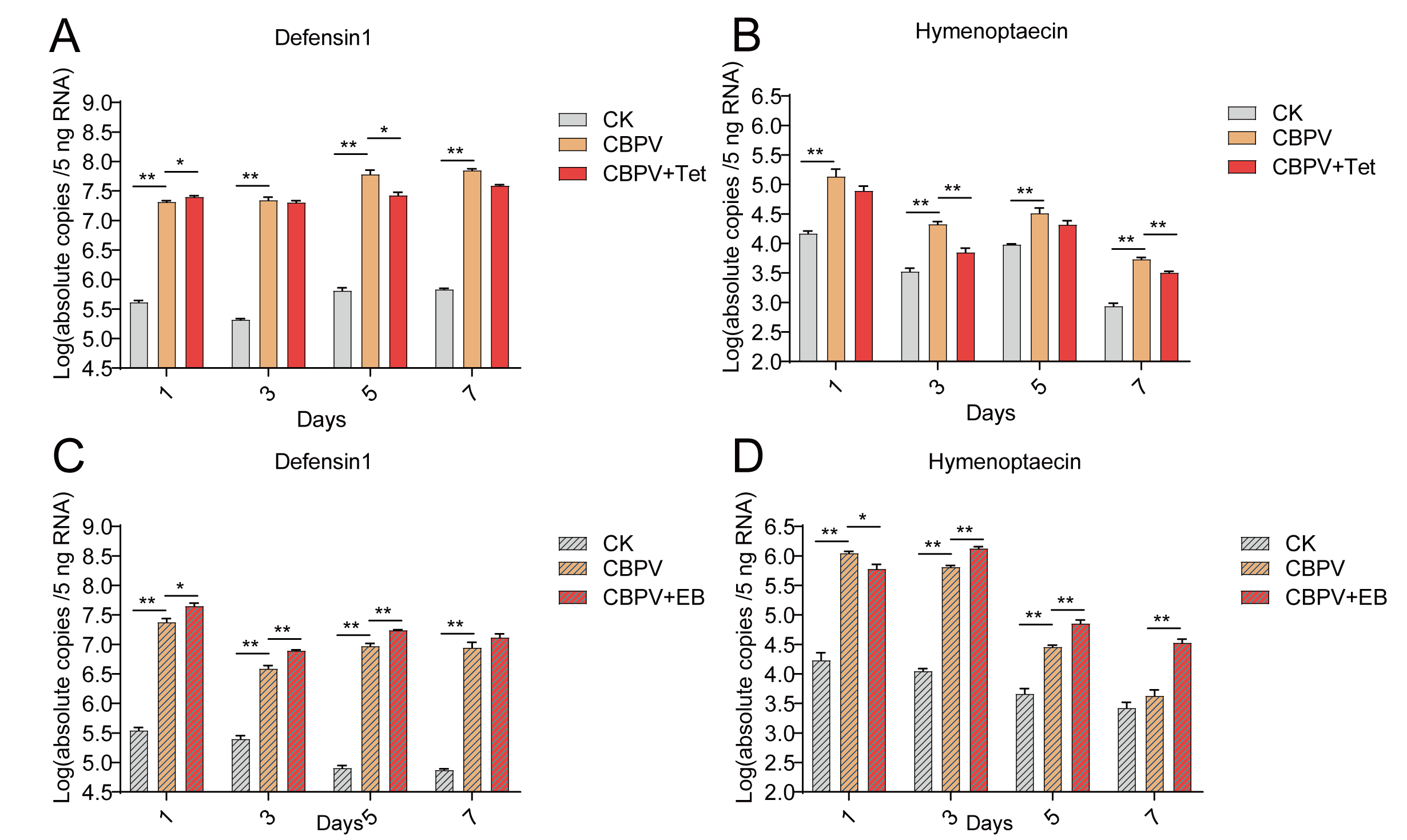
**

**Fig. S7 The comparative analysis of expression level of antimicrobial peptide genes in CBPV-infected *A. mellifera* treated with tetracycline or opportunistic pathogens at days 1, 3, 5, and 7.** (A-B) The expression levels of *defensin1* and *hymenoptaecin* in CBPV-infected *Apis mellifera* treated with tetracycline. (C-D) The expression levels of *defensin1* and *hymenoptaecin* in CBPV-infected *Apis mellifera* treated with opportunistic pathogens. Tet: tetracycline treatment; EB: opportunistic pathogens *Enterobacteria* treatment.

**
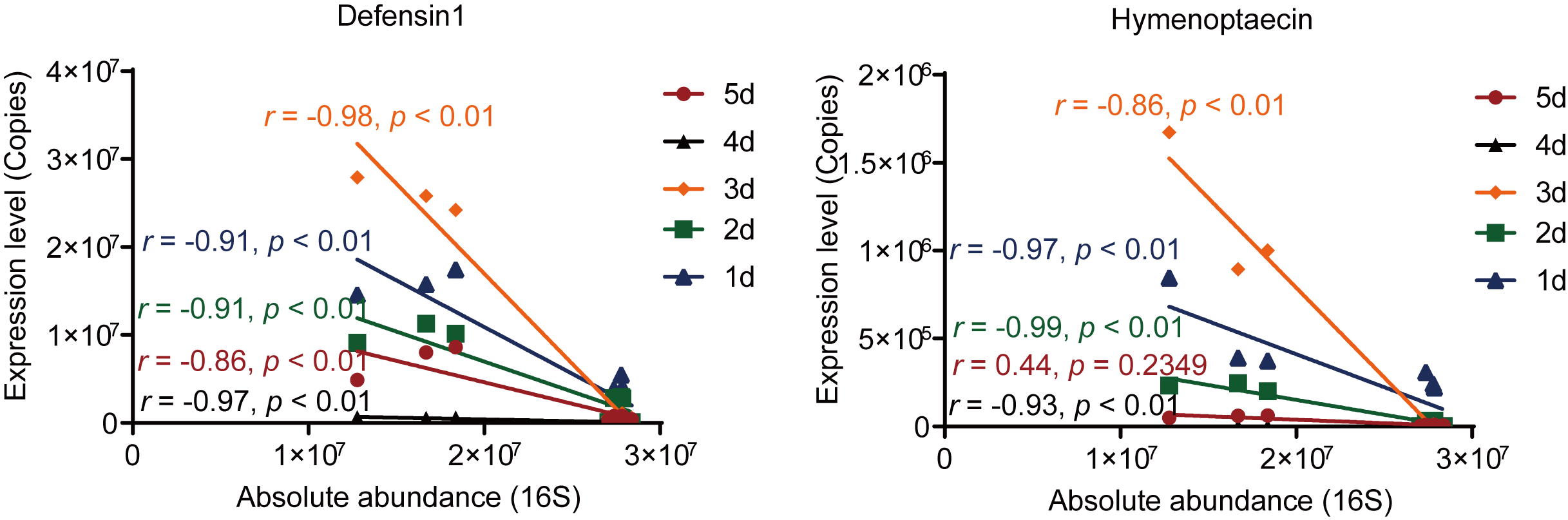
**

**Fig. S8 The correlation analysis between the expression level of AMPs and total bacterial abundance.** (A) Correlation between the expression level of *defensin1* and total bacterial abundance. (B) Correlation between the expression level of *hymenoptaecin* and total bacterial abundance.

**
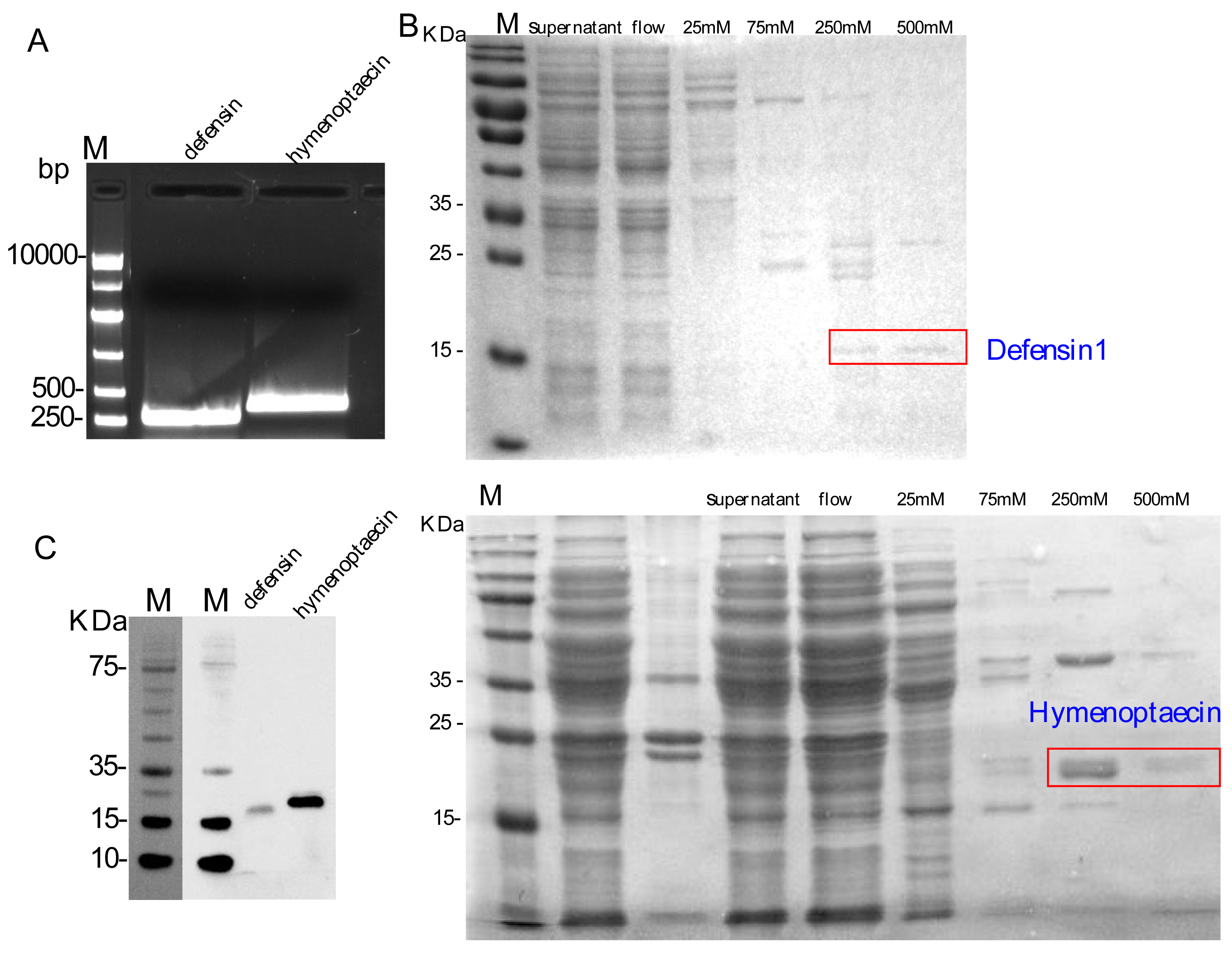
**

**Fig. S9 The expression and purification of AMPs proteins.** (A) The amplification of *defensin1* and *hymenoptaecin*. (B) The purified Defensin1 and Hymenoptaecin proteins were separated by SDS-PAGE. (C) The purified Defensin1 and Hymenoptaecin proteins were identified by Western blot using HRP Conjugated Anti His-Tag Mouse Monoclonal Antibody.


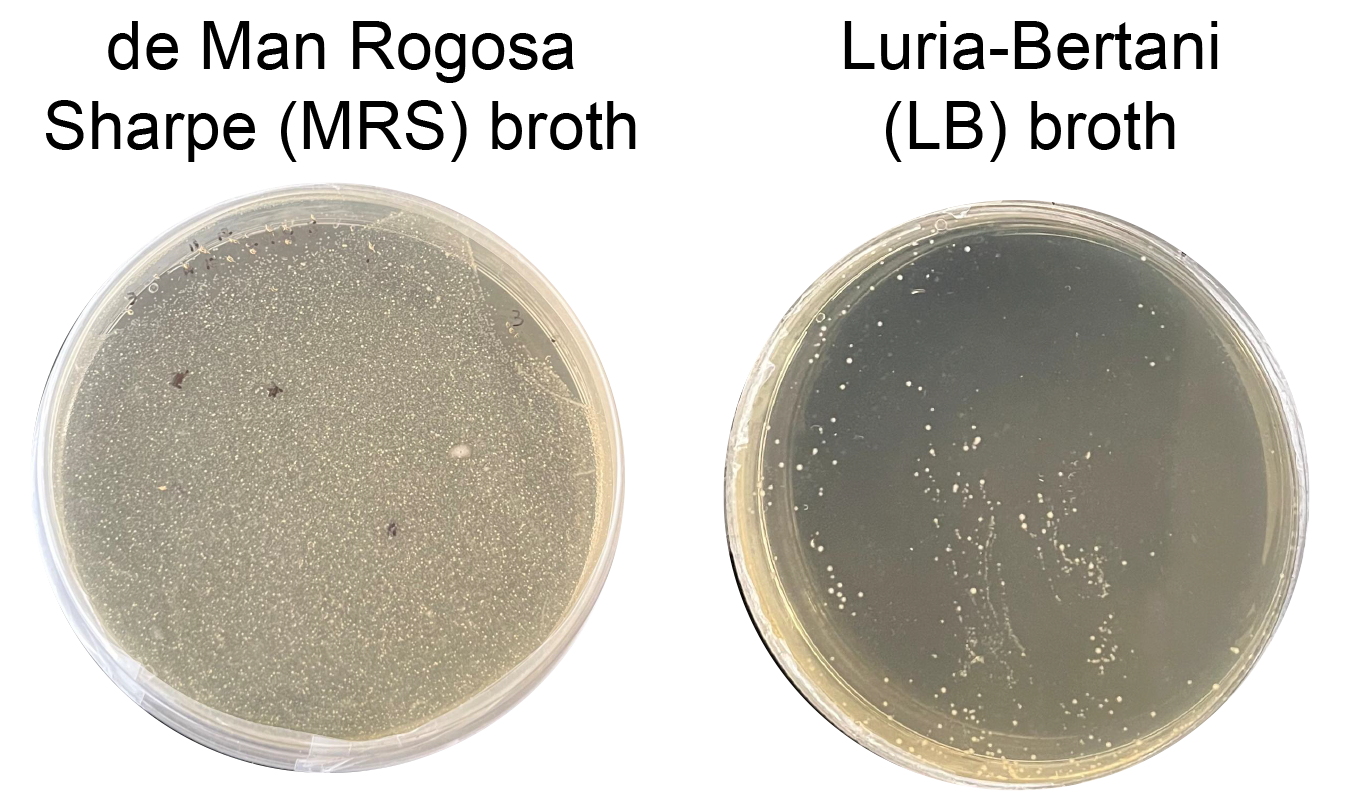


**Fig. S10 The representative MRS/LB plates showed honey bees intestinal bacteria strains were isolated.**

**Supplementary tables**

**Table S1. Primers used to detect honey bee common viruses and bacterial diseases as well as the amplification of AMPs**.

| Viruses/genes | The full name | Primer sequence (5’–3’) | Amplification length | Reference |
| --- | --- | --- | --- | --- |
| IAPV | Israeli acute paralysis virus | F:AGACA CAATCACGGACCTCAC | 475 | [22] |
|  |  | R:AGATTT GTCTGTCT CCAGTG CACAT |  |  |
| DWVa | Deformed wing virus_a | F: TACTAGTGCTGGTTTTCCTTT | 155 | [22, 23] |
|  |  | R: CTCATTAACTGTGTCGTTGAT |  |  |
| CBPV | Chronic bee paralysis virus | F:TCAGACAC GAATCT GATTAT TG | 570 | [24] |
|  |  | R:ACTACTAGAAACTCGTCGCTT CG |  |  |
| BQCV | Black queen cell virus | F:TGGTCAGCT CCCACTACCTTA AAC | 700 | [25] |
|  |  | R:GCAACAAGAAGAAACGTA AA CAC |  |  |
| ABPV | Acute bee paralysis virus | F:TTA TGT GTC CAG AGA CTG TAT | 900 | [25] |
|  |  | R:GCT CCT ATT GCT CGG TTT TTC |  |  |
| CSBV | Chinese sacbrood virus | F:CCTGGGAAGTTTGCTAGTATTTACG | 161 | [26] |
|  |  | R:CCTATCACATCCATCTGGGTCAG |  |  |
| SBV | Sacbrood virus | F:ATA TAC GGT GCG AGA ACT GC | 879 | [27] |
|  |  | R:CTC GGT AAT AAC GCC ACT GT |  |  |
| DWVc | Deformed wing virus_c | F:TACTAGTGCTGGTTTTCCTTT | 152 | [28] |
|  |  | R:ATAAGTTGCGTGGTTGAC |  |  |
| KV | KaKugo virus | F:GATATGACTGTATCCTCCATAGCATCTC | 396 | This study |
|  |  | R:GTATGAAACATATGGCACCTCAAAAG |  |  |
| ALPV | Aphid lethal paralysis virus | F:GCGTACCATACTACTCACCATATTTAT | 140 | This study |
|  |  | R:AGTTAATCCATAAAGTGCAATCTACAA |  |  |
| AFB | American foulbrood | F:GCTCTGTTGCCAAGGAAGAA | 451 | [28] |
|  |  | R:AGGCGGAATGCTTACTGTGT |  |  |
| EFB | European foulbrood | F: GAAGAGGAGTTAAAAGGCGC | 831 | [28] |
|  |  | R: TTATCTCTAAGGCGTTCAAAGG |  |  |
| *defensin 1* | *defensin 1* | F:ATGGGTCGCGGATCCGAATTCATGAAAATCTATTTTATTGT | 288 | This study |
|  |  | R:TTGTCGACGGAGCTCGAATTCTTAACCGAAACGTTTGTC |  |  |
| *hymenoptaecin* | *hymenoptaecin* | F:ATGGGTCGCGGATCCGAATTCATGAAATTCATCGTGTTG | 390 | This study |
|  |  | R:TTGTCGACGGAGCTCGAATTCTCAAAATCTAAATCCACCAT |  |  |

**Table S2. List of primers for qPCR.**

| Gene name | Gene symbol | GenBank  accession no. | Primer sequence (5’–3’) | Amplicon  size (bp) |
| --- | --- | --- | --- | --- |
| chronic bee paralysis virus | CBPV | MF175173.1 | F:GGCACCTCAAGATCGTCCAAGTTAC | 139 |
|  |  |  | R:ACGGAGATGGTGACCTGGTATGG |  |
| *toll* | *toll* | XM_016911914.1 | F:TAGAGTGGCGCATTGTCAAG | 167 |
|  |  |  | R:ATCGCAATTTGTCCCAAAAC |  |
| *peptidoglycan recognition protein S2* | *Pgrp-s2* | NM_001163716.1 | F:TAATTCATCATTCGGCGACA | 171 |
|  |  |  | R:TGTTTGTCCCATCCTCTTCC |  |
| *hymenoptaecin* | *hymenoptaecin* | NM_001011615.1 | F:CTCTTCTGTGCCGTTGCATA | 200 |
|  |  |  | R:GCGTCTCCTGTCATTCCATT |  |
| *defensin 1* | *defensin 1* | NM_001011616.2 | F:AAGAACGTGCCGACAGACAT | 121 |
|  |  |  | R:TCGCAATGACCTCCAGCTTT |  |
| *Defensin 2* | *Defensin 2* | NM_001011638.1 | F:ATTCCAACGAACCGCTGCTA | 150 |
|  |  |  | R:CGCCATTTCTGCAACTACCG |  |
| *Abaecin* | *Abaecin* | NM_001011617.1 | F:TATCTTCGCACTACTCGCCAC | 120 |
|  |  |  | R:TTCGGATTGAATGGTCCCTGA |  |
| *Lysozyme 1* | *Lysozyme 1* | XM_026444741.1 | F:GTTCCAGAGGACATAGCGGG | 120 |
|  |  |  | R:ACGCCTTGAACCCTTCCAAA |  |
| *Lysozyme 2* | *Lysozyme 2* | XM_393161.7 | F:GCATATTCTTCCGCGCATTGT | 289 |
|  |  |  | R:CGAAATGGTCCGCAAACAGA |  |
| *Apisimin* | *Apisimin* | NM_001011582.1 | F:TGCGTAGCCATGTTGGTCAG | 123 |
|  |  |  | R:GTTGGCACCAGACACGATAGA |  |

**Table S3. Standard curves for absolute quantification of CBPV load and immune genes.**

| Genes | The equation of standard curves | Amplification efficiency(E%) | Regression  Coefficient(R^2^) | Tm(℃) |
| --- | --- | --- | --- | --- |
| CBPV | y=－3.22x+35.36 | 102.12 | 0.996 | 84.5 |
| *Toll* | y=－3.2x+31.38 | 105.21 | 0.996 | 80.0 |
| *PGRP-S2* | y=－3.2x+36.22 | 105.23 | 0.998 | 80.5 |
| *Hymenoptaecin* | y=-3.31x+34.42 | 100.35 | 0.999 | 82.0 |
| *Defensin1* | y=－3.23x+36.53 | 106.28 | 1 | 80.5 |

Dataset S4 (separate Table file). The relative abundance of gut microbiome in bees after CBPV infection.

Dataset S5 (separate Table file). The relative abundance of KEGG pathway in gut microbiome of bees after CBPV infection.

Dataset S6 (separate Table file). The relative abundance of CAZy function annotation in gut microbiome of bees after CBPV infection.

Dataset S7 (separate Table file). The FPKM values of expressed genes in bees after CBPV infection.

Dataset S8 (separate Table file). The differentially expressed genes in blue and brown model.

**References**

[1] Rusenova N, Parvanov P, Stanilova S. Detection of *paenibacillus larvae* spores in honey by conventional pcr and its potential for american foulbrood control. *Bulgarian.* J Vet Med. 2018; 22 (163-170).

[2] Diao Q, Li B, Zhao H, et al. Enhancement of chronic bee paralysis virus levels in honeybees acute exposed to imidacloprid: A Chinese case study. Sci Total Environ. 2018;630:487-494.

[3] Kleijn D, Winfree R, Bartomeus I, et al. Delivery of crop pollination services is an insufficient argument for wild pollinator conservation. Nat Commun. 2016;7:10841.

[4] Fischer AH, Jacobson KA, Rose J, Zeller R. Hematoxylin and eosin staining of tissue and cell sections. CSH Protoc. 2008;2008:pdb.prot4986.

[5] Kešnerová L, Mars RAT, Ellegaard KM, Troilo M, Sauer U, Engel P. Disentangling metabolic functions of bacteria in the honey bee gut. PLoS Biol. 2017;15(12):e2003467.

[6] Luo R, Liu B, Xie Y, Li Z, Huang W, Yuan J, et al. Erratum: SOAPdenovo2: an empirically improved memory-efficient short-read de novo assembler. Gigascience. 2015; 4:30. doi:

[7] Qin N, Yang F, Li A, Prifti E, Chen Y, Shao L, et al. Alterations of the human gut microbiome in liver cirrhosis. Nature. 2014;513(7516):59-64.

[8] Feng Q, Liang S, Jia H, Stadlmayr A, Tang L, Lan Z, et al. Gut microbiome development along the colorectal adenoma-carcinoma sequence. Nat Commun. 2015;6:6528.

[9] Buchfink B, Xie C, Huson DH. Fast and sensitive protein alignment using DIAMOND. Nat Methods. 2015;12(1):59-60.

[10] Oh J, Byrd AL, Deming C, Conlan S; NISC Comparative Sequencing Program, Kong HH, Segre JA. Biogeography and individuality shape function in the human skin metagenome. Nature. 2014;514(7520):59-64.

[11] Huson DH, Mitra S, Ruscheweyh HJ, Weber N, Schuster SC. Integrative analysis of environmental sequences using MEGAN4. Genome Res. 2011;21(9):1552-60.

[12] Segata N, Izard J, Waldron L, Gevers D, Miropolsky L, Garrett WS, Huttenhower C. Metagenomic biomarker discovery and explanation. Genome Biol. 2011;24;12(6):R60.

[13] Kanehisa M, Goto S, Sato Y, Kawashima M, Furumichi M, Tanabe M. Data, information, knowledge and principle: back to metabolism in KEGG. Nucleic Acids Res. 2014;42(Database issue):D199-205.

[14] Elsik CG, Worley KC, Bennett AK, et al. Finding the missing honey bee genes: lessons learned from a genome upgrade. BMC Genomics. 2014;15:86.

[15] Trapnell C, Williams BA, Pertea G, et al. Transcript assembly and quantification by RNA-Seq reveals unannotated transcripts and isoform switching during cell differentiation. Nat Biotechnol. 2010;28(5):511-515.

[16] Liu K, Chen S, Lu R. Identification of important genes related to ferroptosis and hypoxia in acute myocardial infarction based on WGCNA. Bioengineered. 2021;12(1):7950-7963.

[17] Raymann K, Shaffer Z, Moran NA. Antibiotic exposure perturbs the gut microbiota and elevates mortality in honeybees. PLoS Biol. 2017;15(3):e2001861.

[18] Jin Y, Zeng Z, Wu Y, Zhang S, Fu Z. Oral exposure of mice to carbendazim induces hepatic lipid metabolism disorder and gut microbiota dysbiosis. Toxicol Sci. 2015;147(1):116-26.

[19] Ohad S, Block C, Kravitz V, Farber A, Pilo S, Breuer R, Rorman E. Rapid identification of *Enterobacter hormaechei* and *Enterobacter cloacae* genetic cluster III. J Appl Microbiol. 2014;116(5):1315-21.

[20] Dosch C, Manigk A, Streicher T, Tehel A, Paxton RJ, Tragust S. The gut Microbiota can provide viral tolerance in the honey bee. Microorganisms. 2021;9(4):871.

[21] Mohkam M, Nezafat N, Berenjian A, Mobasher MA, Ghasemi Y. Identification of *Bacillus* probiotics isolated from soil rhizosphere using 16S rRNA, recA, rpoB gene sequencing and RAPD-PCR. Probiotics Antimicrob Proteins. 2016;8(1):8-18.

[22] Maori E, Lavi S, Mozes-Koch R, Gantman Y, Peretz Y, Edelbaum O, Tanne E, Sela I. Isolation and characterization of Israeli acute paralysis virus, a dicistrovirus affecting honeybees in Israel: evidence for diversity due to intra- and inter-species recombination. J Gen Virol. 2007;88(Pt 12):3428-3438.

[23] Kevill JL, Highfield A, Mordecai GJ, Martin SJ, Schroeder DC. ABC assay: method development and application to quantify the role of three DWV master variants in overwinter colony losses of European honey bees. Viruses. 2017;9(11):314.

[24] Li B, Deng S, Yang D, Hou C, Diao Q. Complete sequences of the RNA 1 and RNA 2 segments of chronic bee paralysis virus strain CBPV-BJ detected in China. Arch Virol. 2017;162(8):2451-2456.

[25] Benjeddou M, Leat N, Allsopp M, Davison S. Detection of acute bee paralysis virus and black queen cell virus from honeybees by reverse transcriptase pcr. Appl Environ Microbiol. 2001;67(5):2384-7.

[26] Mingxiao M, Jinhua L, Yingjin S, Li L, Yongfei L. TaqMan MGB probe fluorescence real-time quantitative PCR for rapid detection of Chinese Sacbrood virus. PLoS One. 2013;8(2):e52670.

[27] Hou C, Rivkin H, Slabezki Y, Chejanovsky N. Dynamics of the presence of israeli acute paralysis virus in honey bee colonies with colony collapse disorder. Viruses. 2014;6(5):2012-27.

[28] Deng Y, Yang S, Zhao H, Luo J, Yang W, Hou C. Antibiotics-induced changes in intestinal bacteria result in the sensitivity of honey bee to virus. Environ Pollut. 2022;314:120278.
